# Neuronal Computation Underlying Inferential Reasoning in Humans and Mice

**DOI:** 10.1016/j.cell.2020.08.035

**Published:** 2020-10-01

**Authors:** Helen C. Barron, Hayley M. Reeve, Renée S. Koolschijn, Pavel V. Perestenko, Anna Shpektor, Hamed Nili, Roman Rothaermel, Natalia Campo-Urriza, Jill X. O’Reilly, David M. Bannerman, Timothy E.J. Behrens, David Dupret

**Affiliations:** 1 Medical Research Council Brain Network Dynamics Unit, Nuffield Department of Clinical Neurosciences, University of Oxford, Mansfield Road, Oxford OX1 3TH, UK; 2 Wellcome Centre for Integrative Neuroimaging, University of Oxford, FMRIB, John Radcliffe Hospital, Oxford OX3 9DU, UK; 3 Department of Experimental Psychology, University of Oxford, 15 Parks Road, Oxford OX1 3AQ, UK; 4 The Wellcome Trust Centre for Neuroimaging, Institute of Neurology, University College London, London WC1N 3BG, UK

**Keywords:** inference, memory, hippocampus, humans, mice, sharp-wave ripple, prospective code, cognitive short-cut, cognitive map

## Abstract

Every day we make decisions critical for adaptation and survival. We repeat actions with known consequences. But we also draw on loosely related events to infer and imagine the outcome of entirely novel choices. These inferential decisions are thought to engage a number of brain regions; however, the underlying neuronal computation remains unknown. Here, we use a multi-day cross-species approach in humans and mice to report the functional anatomy and neuronal computation underlying inferential decisions. We show that during successful inference, the mammalian brain uses a hippocampal prospective code to forecast temporally structured learned associations. Moreover, during resting behavior, coactivation of hippocampal cells in sharp-wave/ripples represent inferred relationships that include reward, thereby “joining-the-dots” between events that have not been observed together but lead to profitable outcomes. Computing mnemonic links in this manner may provide an important mechanism to build a cognitive map that stretches beyond direct experience, thus supporting flexible behavior.

## Highlights

• Inferential decisions engage the hippocampus in humans and mice
• During inference, a hippocampal prospective code draws on associative memories
• This hippocampal prospective code preserves the learned temporal statistics
• During rest, hippocampal ripples nest cognitive short-cuts for inferred relations

## Graphical Abstract

**Figure:**
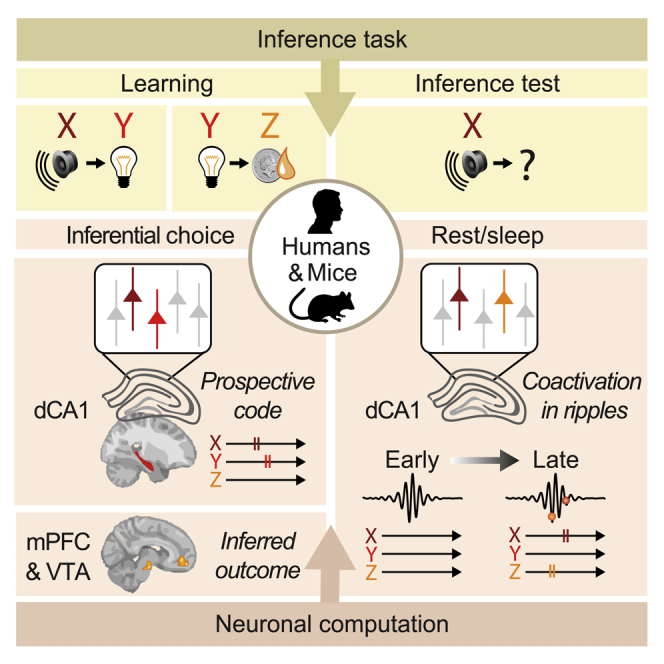

## Teaser

In humans and mice, the hippocampus supports inferential reasoning by computing a prospective code to predict upcoming events, before extracting logical links between discrete events during rest to form a mnemonic short cut for inferred relationships.

## Introduction

When making decisions, we often draw on previous experience. We repeat actions that were profitable in the past and avoid those that led to unwanted consequences. However, we can also make decisions using information we have not directly experienced, by combining knowledge from multiple discrete items or events to infer new relationships. This ability to infer previously unobserved relationships is thought to be critical for flexible and adaptive behavior.

Anatomical lesions in rodents and functional imaging in humans have started to uncover the macroscopic network of brain regions supporting inferential decisions (Bunsey and Eichenbaum, 1996; Hampton et al., 2006; Jones et al., 2012; Nicholson and Freeman, 2000; Preston et al., 2004; Robinson et al., 2014; Wimmer and Shohamy, 2012; Zeithamova et al., 2012a), highlighting the involvement of orbitofrontal, medial prefrontal, perirhinal, and retrosplenial cortices, along with the hippocampus. However, the mechanistic contribution of these regions and the neuronal computation underpinning inference remain unclear.

A potential mechanism for inference involves chaining together memories for discrete events at the time of choice. In this scenario, an inferred outcome is predicted by internally simulating the short-term consequences of each memory in the chain. Retrieval mechanisms of this kind may be described by a family of theories known as model-based reinforcement learning (Daw et al., 2005) that involve a learned model of the world. By constructing predictions for decision outcomes on the fly, such mechanisms capture a hallmark of flexible decision-making. However, this comes with the computational cost of searching through a potentially large number of memories.

To reduce the computational demand associated with inference, events that have not been encountered together in space or time may be linked to form cognitive “short-cuts.” Together with prior memories, such higher-order relationships may form a “relational” or “cognitive map” of the world (Cohen and Eichenbaum, 1993; O’Reilly and Rudy, 2001; Tolman, 1948). The hippocampus has been attributed to holding a cognitive map (O’Keefe and Nadel, 1978), with neuronal representations observed in the spatially tuned activity of pyramidal cells during exploration (Ekstrom et al., 2003; O’Keefe and Dostrovsky, 1971). In addition to representing space, the hippocampus supports memory for past experience (Squire, 1992) and mediates associations between sequential events (Fortin et al., 2002; Schendan et al., 2003). However, while the hippocampus is a suitable candidate to hold internal maps, it remains unclear whether this brain region represents or computes cognitive short-cuts to support inference.

One possibility is that memories for distinct experiences are linked together or even fundamentally restructured during awake rest and sleep (Buckner, 2010; Buzsáki, 2015; Diekelmann and Born, 2010; Foster, 2017; Joo and Frank, 2018). During these quiet periods, hippocampal local-field potentials (LFPs) are characterized by sharp-wave/ripples (SWRs): short-lived, large-amplitude deflections accompanied by high-frequency oscillations (Buzsáki, 2015; Csicsvari et al., 1999). During SWRs, hippocampal cells fire synchronously and their temporally structured spiking can “replay” previous waking experience (Louie and Wilson, 2001; Nádasdy et al., 1999; Wilson and McNaughton, 1994) to support memory and planning (Buzsáki, 2015; Foster, 2017; Joo and Frank, 2018). Growing evidence suggests SWR activity also extends beyond replay of directly experienced information. For instance, hippocampal SWR spiking can anticipate upcoming experience (Dragoi and Tonegawa, 2011; Ólafsdóttir et al., 2015), reorder events according to a trained rule (Liu et al., 2019), or even stitch together spatial trajectories (Gupta et al., 2010; Wu and Foster, 2014). In this manner, we hypothesize that hippocampal SWR activity generates spiking motifs that provide a cellular basis for novel higher-order relationships, thus breaking the constraints imposed by direct experience.

Here, we investigate the neuronal computation underlying inference in the mammalian brain using a cross-species approach. We implement a multi-day inference task and deploy brain recording technologies in both humans and mice to synergize insights gained across species. Namely, we acquire near-whole brain ultra-high field (7T) functional magnetic resonance imaging (fMRI) in humans to identify where inference is computed, before using this finding to inform optogenetic manipulations in mice to test causality.

Using human 7T fMRI and mouse *in vivo* multichannel electrophysiology, we then obtain complementary signatures of inference at the macroscopic and cellular resolution, respectively. By implementing the same analytical framework across species, we show that during inferential choice the hippocampus forecasts mnemonic, temporally structured associations “on-the-fly.” While this prospective code draws on learned experience, in humans the inferred outcome is represented in the medial prefrontal cortex (mPFC) and the putative dopaminergic midbrain. Next, during rest/sleep in mice, neuronal coactivations during hippocampal SWRs increasingly represent inferred relationships that include reward, thus “joining-the-dots” between discrete events. These findings show that the hippocampus supports inference by computing a prospective code to “look ahead” and predict upcoming experience, before extracting “logical” links between events in SWRs. In this manner, the hippocampus may construct a cognitive map that stretches beyond direct experience (O’Keefe and Nadel, 1978; Tolman, 1948), creating new knowledge to facilitate flexible future decisions.

## Results

### Cross-Species Task Design and Behavioral Performance

**Figure 1:**
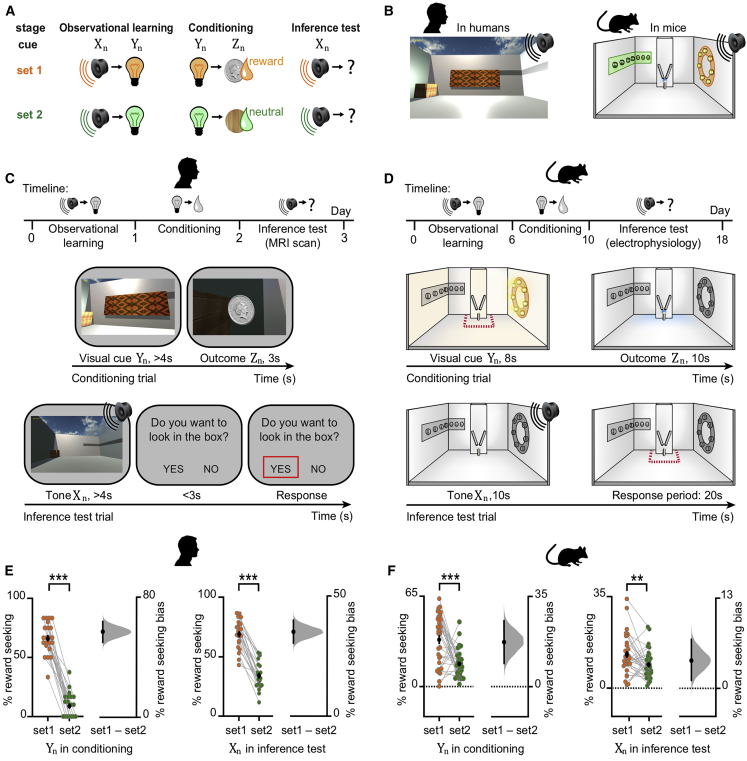
Inference Task Design and Behavioral Performance in Humans and Mice (A) Three-stage inference task. Humans and mice learned to associate auditory cues (Xn) with visual cues (Yn) (“observational learning”); visual cues (Yn) with an outcome (Zn) (“conditioning”), where the outcome was rewarding in set 1 (orange) and neutral in set 2 (green). In the “inference test”, the auditory cues (Xn) were presented in isolation and reward-seeking behavior quantified as a measure of inference from Xn to Zn. (B) Virtual-reality environment used with humans (left), simulating the open-field arena used with mice (right schematic). Outcomes Zn were delivered to a wooden box (humans) or a liquid dispenser (mice). (C and D) Top: timeline for the task in humans (C) and mice (D). Middle: example conditioning trials (schematic). Bottom: example inference test trials (schematic). (C) Middle and bottom: a subset of conditioning trials and all inference test trials were performed inside a 7T scanner. Red square indicates the participant’s response. (D) Middle and bottom: all conditioning and inference test trials were performed within the open-field. Red dotted line delineates outcome area around the dispenser. (E and F) Left of each panel: raw data points for set 1 (orange) and set 2 (green); black dot, mean; black ticks, ±SEM. Right of each panel: behavioral measures of reward-seeking bias shown using bootstrap-coupled estimation (DABEST) plots (Ho et al., 2019). Effect size for the difference between set 1 and 2 (i.e., reward seeking bias), computed from 10,000 bias-corrected bootstrapped resamples (Efron, 2000): black dot, mean; black ticks, 95% confidence interval; filled-curve, sampling-error distribution. (E) Humans exhibited significant reward-seeking bias (percentage of trials where participants wished to visit the wooden box in “set 1” relative to “set 2”) in response to visual cues during conditioning (p < 0.001) and auditory cues during the inference test (p < 0.001). Each data point: average reward-seeking bias of one participant. (F) Mice showed significant reward-seeking bias (percentage of time spent in the outcome area in “set 1” relative to “set 2”) during visual cues in conditioning (p < 0.001; Figures S2A–S2F) and following auditory cues in the inference test (p = 0.005; Figures S2G and S2H). Each data point: average reward-seeking bias of one mouse on a given day. Both humans and mice showed greater reward seeking bias for visual cues Yn (directly paired with Zn), compared to auditory cues Xn (indirectly paired with Zn). See also Figure S1 and Table S4.

**Figure S1:**
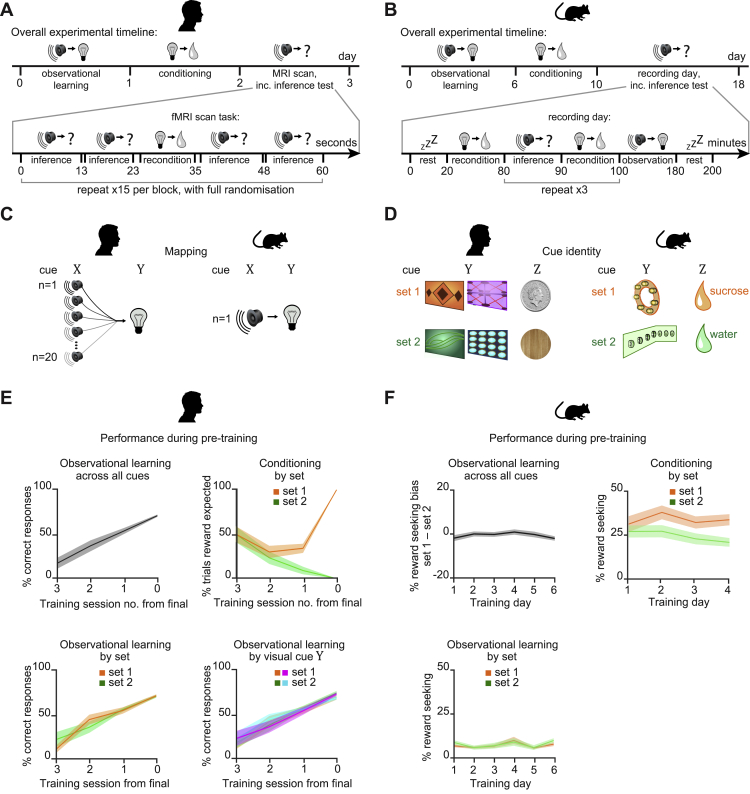
In humans and Mice: Inference Task Design and Pre-training Performance, Related to Figure 1 In humans and mice: (A-B) Structure of the inference task in humans and mice. (A) In humans, the experiment was conducted across 3 days. Participants completed observational learning on day 1, conditioning on day 2, and an MRI scan on day 3. During the MRI scan participants were presented with conditioning trials (“reconditioning”) and inference test trials (Figure 1C), presented in a pseudorandom order. (B) In mice, the inference task was conducted across 18 days. Observational learning was conducted across days 1-6, conditioning across days 7-10, and recordings across days 11-18. Each recording day started with a sleep/rest block, after which mice performed conditioning trials (“reconditioning”). The inference test was delivered in 3 separate blocks that were interleaved by reconditioning blocks to minimize extinction effects. At the end of the final inference test, mice were given additional conditioning trials, before being re-exposed to the observational learning. At the end of the recording day mice were recorded during a second sleep/rest block. The two sleep/rest periods were excluded from recording days in mice implanted with optic fibers in the absence of tetrodes. (C) In humans (left) there was a many-to-one mapping between auditory and visual cues. In mice (right) there was a one-to-one mapping between auditory and visual cues. (D) In humans (left) there were four possible visual cues, two in set 1 and two in set 2, which mapped onto two possible outcomes, a monetary reward or a neutral wood-chip. The silver coins or neutral wood-chips could be collected from the wooden box in the corner of the room. In mice (right) there were two possible visual cues, one in set 1 and one in set 2, which mapped onto two possible outcomes, a sucrose (rewarding) or water (neutral) drop delivered to the liquid dispenser. (E-F) Behavioral performance in humans and mice during the pre-training observational learning and conditioning stages of the task (mean ± SEM). (E) In humans. Participants performed the observational learning task until they showed accurate recall of at least 50% of all auditory-visual pairs (left). Participants performed the conditioning task until they reached 100% accuracy on all visual-outcome associations (right). (F) In mice. The observational learning was conducted across 6 days. As expected, mice did not show a reward-seeking bias for cues in set 1 or 2 during this stage of the pretraining (left). Reward-seeking during the observational learning was defined as the percentage time spent in the outcome area in the 20 s period after the auditory cue (Xn) (Figure 1D). After day 3 of the observational learning the time spent in the outcome area following cues in both set 1 and set 2 increased, coinciding with mice being food restricted to 90% their original body weight. The conditioning was conducted across 4 days, during which reward-seeking bias for cues in set 1 compared to set 2 increased (right), indicating that mice learned to associate the visual cues (Yn) with the relevant outcome cues (Zn). Reward-seeking during the conditioning pre-training was defined as the percentage time spent in outcome area during outcome (Zn) availability.

We designed a three-stage task (Figure 1A) that leveraged a sensory preconditioning paradigm (Brogden, 1939) while permitting recordings of brain activity in humans and mice. To match the paradigm across species, we trained human participants in a virtual-reality environment simulating the open-field arena used with mice (Figure 1B). The inference task was performed across multiple days (Figures 1C and 1D). In the first stage, we exposed subjects to pairs of sensory stimuli, with each pair n including an auditory cue Xn that signaled contiguous presentation of a visual cue Yn (Figure 1A; Xn→Yn “observational learning”). In the second stage, we re-exposed subjects to the visual cues Yn, each of which now predicted delivery of either a rewarding (set 1 stimuli) or neutral (set 2 stimuli) outcome Zn (Figure 1A; Yn→Zn “conditioning”). Rewarding outcomes were virtual silver coins for humans (exchangeable for a real monetary sum) and drops of sucrose for mice. Neutral outcomes were (non-exchangeable) woodchips for humans and drops of water for mice. In humans, we included a many-to-one mapping between task cues (Figures S1C and S1D), to further dissociate cue-specific representations. Importantly, auditory cues Xn were never paired with outcomes Zn, providing an opportunity to assess evidence for an inferred relationship between these indirectly related stimuli. Accordingly, in the final stage, we presented auditory cues Xn in isolation, without visual cues Yn or outcomes Zn, and we measured evidence for inference from Xn to Zn by quantifying reward-seeking behavior (Figures 1A, Xn→? “inference test”, 1C, and 1D).

**Figure S2:**
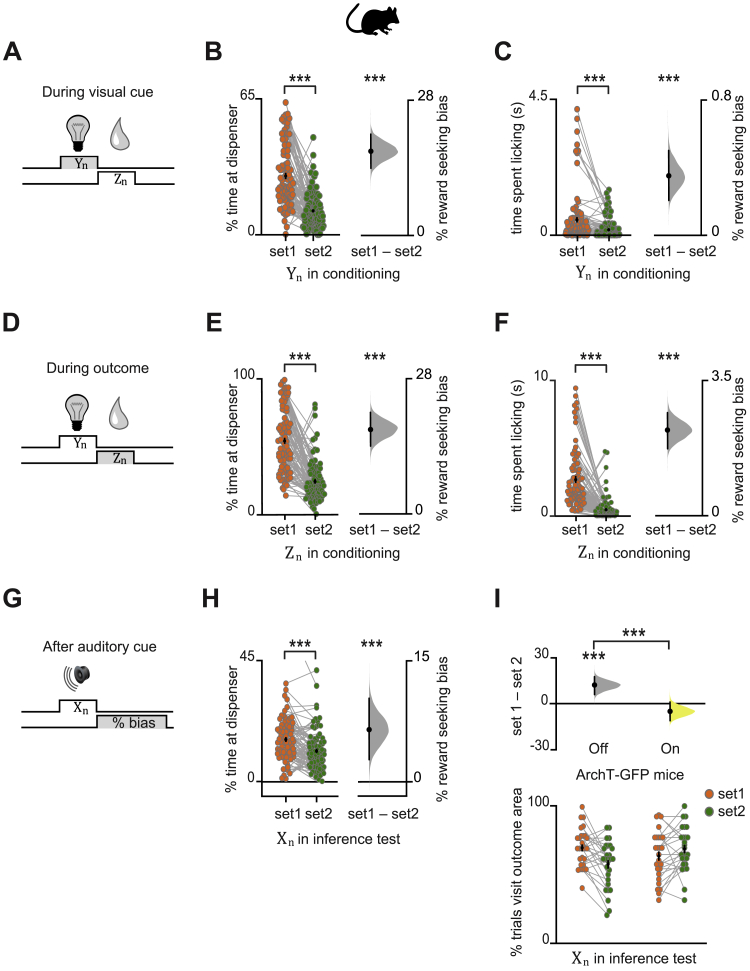
Behavior across All Mice during Conditioning and Inference Test, Related to Figures 1 and 2 In mice: (A-I) Data Analysis with Bootstrap-coupled ESTimation (DABEST) plots (Ho et al., 2019) used to visualize the effect size of behavioral measures of reward seeking bias. Raw data points are shown for set 1 and set 2 in orange and green respectively, with mean ± SEM shown by *black-dot* and *black-ticks* respectively. The effect size for the difference between set 1 and 2 (i.e., reward seeking bias) is shown as a sampling-error distribution, computed from 10,000 bias-corrected bootstrapped resamples (Efron, 2000): *black-dot*, mean; *black-ticks*, 95% confidence interval; *filled-curve*, sampling-error distribution; *yellow*, laser On; *gray*, laser Off. (A-H) Across recording days in all mice. (A-C) During visual cues (Yn) in conditioning, greater reward-seeking bias was observed for ‘set 1’ relative to ‘set 2’, with reward-seeking bias defined as the percentage time spent in the outcome area (*B*, p < 0.001), or defined as time spent licking in anticipation of an outcome (*C*, p < 0.001). Each data point shows the average reward-seeking bias of a single mouse on a given day. (D-F) After visual cues in conditioning, during the outcome period (Zn), greater reward-seeking bias was observed for ‘set 1’ relative to ‘set 2’, with reward-seeking bias defined as the percentage of time spent in the outcome area (*E*, p < 0.001), or defined as time spent licking the outcome (*F*, p < 0.001). Each data point shows the average reward-seeking bias of a single mouse on a given day. (G-H) Following auditory cues (Xn) in the inference test, greater reward-seeking bias was observed for ‘set 1’ relative to ‘set 2’, with reward-seeking bias defined as the percentage time spent in the outcome area (p < 0.001; with one data point at coordinates [1,2;0.56,0.35] off the display). (I) In ArchT-GFP mice, dCA1 light delivery during auditory cues (Xn) in the inference test impaired reward-seeking bias observed for ‘set 1’ relative to ‘set 2’ (Figure 2I). This effect was further observed using alternative measures of reward seeking bias shown here, where reward seeking bias is defined as the percentage of trials with visit to outcome area following the auditory cue (laser Off p < 0.001; laser On p = 0.105; laser Off – laser On: t_54_ = 3.86, p < 0.001).

During the conditioning, both humans and mice were trained to show higher levels of reward-seeking behavior during visual cues Yn in set 1 relative to set 2. As expected, in response to Yn, subjects successfully anticipated the relevant outcome Zn prior to its delivery (Figures 1C–1F, S1A–S1F, and S2A–S2F).

During the inference test, both humans and mice showed significantly greater reward-seeking behavior in response to auditory cues Xn in set 1 relative to set 2 (Figures 1C–1F, S1A, S1B, S2G, and S2H). Therefore, despite never directly experiencing outcome Zn in response to auditory cues Xn, both species showed behavioral evidence for an inferred relationship between these stimuli.

### The Hippocampus Is Engaged during Inference: Macroscopic Network in Humans

**Figure 2:**
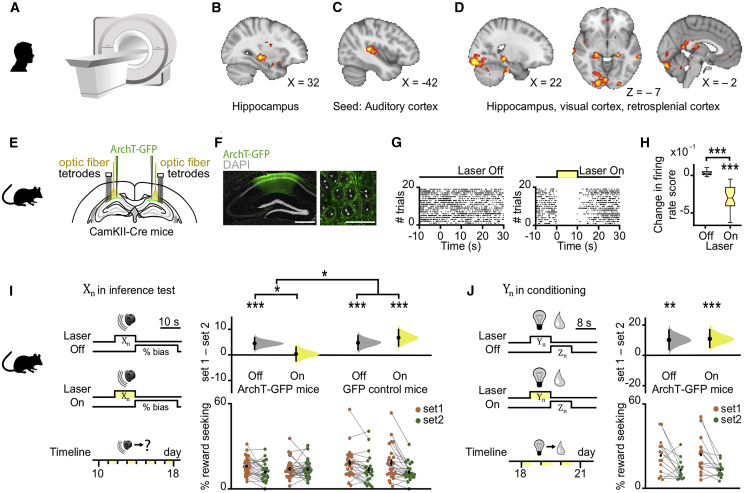
Macroscopic Inference Network in Humans and the Necessary Contribution of dCA1 to Inference in Mice (A) 7T fMRI used to measure the BOLD signal during the inference task (Figure 1C). (B) Significant right hippocampal BOLD signal during correct inference (“correct” – “incorrect” inference: right, t_21_ = 4.15, p = 0.022; left, t_21_ = 2.80, p = 0.221; Figure S3A; Table S1). (C) Significant BOLD signal in auditory cortex during inference test trials (“inference trials” – “conditioning trials”: t_21_ = 14.76, p < 0.001). (D) Psychological-physiological interaction showing differential co-activation with auditory cortex (seed region, C, Figure S3B) on correct versus incorrect inference trials (hippocampus: t_21_ = 4.23, p = 0.015; and other regions: retrosplenial cortex: t_21_ = 3.88, p = 0.012; visual cortex: t_21_ = 4.77, p < 0.001; Table S2). (E–J) In mice. Yellow, laser on; gray, laser off. (E) Schematic: ArchT-GFP viral injections, optic fibers, and tetrodes targeting dCA1 of CamKII-Cre mice for ensemble recording and manipulation. (F) dCA1 (green) ArchT-GFP expression. Scale bar, 500 μm (left), 50 μm (right). (G) Raster plot showing photo-silencing of spiking activity for an example dCA1 pyramidal cell from an ArchT-GFP mouse. (H) Light-induced changes in firing rate for simultaneously recorded dCA1 pyramidal cells in an example ArchT-GFP mouse (laser on: t_30_ = −10.86, p < 0.001; laser off – laser on: t_30_ = 10.88, p < 0.001). Rate changes expressed for each cell as the differences between laser on and laser off firing over the sum (scores; center line, median; box limits, upper and lower quartiles; whiskers, 1.0× interquartile range). (I and J) Left panel: schematic of light delivery. Bottom right panel: raw data points for set 1 (orange) and set 2 (green); black dot, mean; black ticks, ± SEM. Top right panel: behavioral measures of reward seeking bias shown using DABEST plots, as in Figures 1E and 1F. (I) dCA1 light delivery during auditory cues Xn in the inference test impaired reward-seeking bias in ArchT-GFP mice (set 1 – set 2: laser off p < 0.001; laser on p = 0.794; laser off – laser on: t_54_ = 2.25, p = 0.029; alpha set to 0.05; Figure S2I) but not in GFP control mice (set 1 – set 2: laser off p < 0.001; laser on p < 0.001; laser off – laser on: t_46_ = −0.85, p = 0.399; alpha set to 0.05). The significant reward-seeking biases (ArchT-GFP laser off; GFP control laser off and on) remained significant with Bonferroni correction for four comparisons, alpha set to 0.013. A significant interaction was also observed between the ArchT-GFP and GFP control mice (group × laser interaction, two-way ANOVA, F_1,100_ = 4.42, p = 0.038). (J) dCA1 optogenetic silencing during visual cues Yn, presented after the inference task was complete, did not impair reward-seeking bias in ArchT-GFP mice (set 1 – set 2: laser off p = 0.003; laser on p < 0.001; laser off – laser on: t_26_ = −0.14, p = 0.891). See also Table S4.

**Figure S3:**
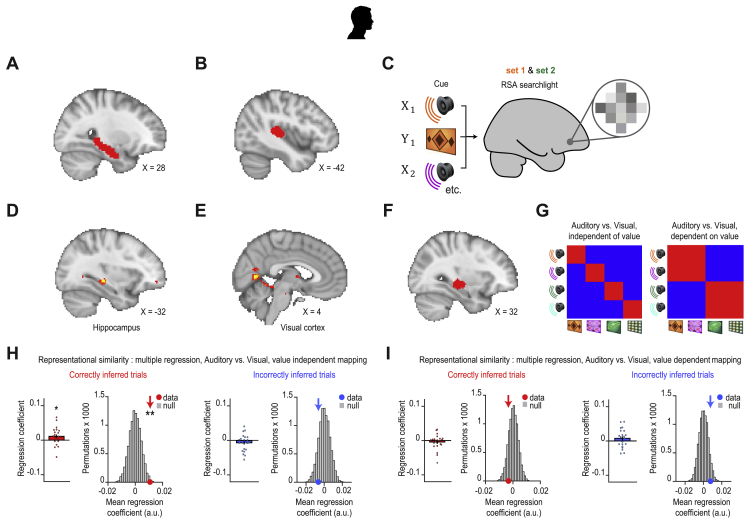
Regions of Interest and RSA during the Inference Test in Humans, Related to Figures 2 and 4 In humans: (A) An anatomical ROI in the hippocampus. This ROI was used to correct for multiple comparisons across the hippocampal volume (Figure 2B). (B) An ROI in auditory cortex was defined from a contrast comparing BOLD signal during inference test trials to conditioning trials (Figure 2C). This ROI was then used as a seed region for a Psychological-Physiological Interaction analysis which identified brain regions that differentially co-activate with auditory cortex across correct and incorrect trials in the inference test (Figure 2D). (C) During correctly inferred trials in the inference test, searchlight RSA was applied to fMRI data in humans to identify brain regions showing representational similarity between auditory cues Xn and visual cues Yn. A model representational similarity matrix (Figure 4E) that mapped the auditory-to-visual associations learned during the inference task was compared with the activity patterns across voxels extracted from each searchlight, using a summary statistic estimated as follows: [average within association XnYn correlations from RSM main diagonal] – [average between association XnYm correlations from RSM off-diagonals]. (D) The searchlight analysis described in (*C*) revealed significant auditory-visual mappings in the hippocampus (t_21_ = 4.76, p = 0.025; peak-level FWE corrected using small-volume correction method), as observed in Figure 4. (E) The searchlight analysis described in (*C*) further revealed significant auditory-visual mappings in the visual cortex (t_21_ = 4.94, p = 0.012, FWE whole-brain corrected at the cluster-level). T-statistic maps are thresholded at p < 0.01 uncorrected for visualization purposes only. (F) In humans, RSA was applied to BOLD signal extracted from a region of interest (ROI) in the hippocampus, defined from the univariate contrast reported in Figure 2B, thresholded at p < 0.01 uncorrected. (G) Applying RSA to hippocampal activity we modeled the mappings from Xn in the inference test to Yn in conditioning, both independent of the value of the associated Zn (left), and dependent upon the value of the associated Zn (right). We then used multiple regression to regress the data onto these two models, to assess evidence for prospective representation of visual cues Yn over and above representation of the value associated with the visual cues. (H) Using multiple regression described in *G*, during correct inference, activity patterns in the hippocampus significantly predicted mappings from Xn to Yn, independent of the value of the associated Zn cues (correct and incorrect inference: Z_21_ = 2.01, p = 0.022 and Z_21_ = −1.07, p = 0.858; mean ± SEM). The group mean was further compared against a null distribution generated by permuting the identity labels assigned to the auditory cues Xn (correct and incorrect inference: p = 0.006 and p = 0.854). (I) Using multiple regression described in *G*, during correct inference, activity patterns in the hippocampus did not significantly predict mappings from Xn to Yn, dependent upon the value of the associated Zn cues (correct and incorrect inference: Z_21_ = −0.39, p = 0.652 and Z_21_ = 1.23, p = 0.109; mean ± SEM). The group mean was further compared against a null distribution generated by permuting the identity labels assigned to the auditory cues Xn (correct and incorrect inference: p = 0.839 and p = 0.070). Thus, during inferential choice hippocampal activity in humans prospectively represents visual cues Yn over and above representation of the value of the associated Zn cues.

To identify where inference is computed, we took advantage of near-whole brain imaging in humans using 7T fMRI (Figure 2A) to measure the blood oxygen level dependent (BOLD) signal during the inference test and conditioning trials. We used two independent analyses. First, by comparing correct and incorrect trials in the inference test, we observed significantly higher BOLD signal in the hippocampus during correct trials (Figures 2B and S3A; Table S1), consistent with animal lesion studies and previous human imaging (Bunsey and Eichenbaum, 1996; Gilboa et al., 2014; Preston et al., 2004). Second, by taking the auditory cortex as a seed, a region showing elevated BOLD signal across all inference test trials (Figures 2C and S3B), we identified brain regions that co-activate with auditory cortex differentially across correct and incorrect trials. Again, we observed a significant effect in the hippocampus, along with a broader network including retrosplenial and visual cortices (Figure 2D; Table S2). These results suggest hippocampal activity is modulated during correct inference, together with brain regions important for memory and the processing of relevant sensory cues.

### The Hippocampus Is Required for Inferential Choice: Optogenetic Silencing in Mice

We next used these findings in humans to guide neuronal silencing in mice, leveraging the cellular and temporal precision of optogenetic tools (Deisseroth, 2011) to test the causal contribution of hippocampal activity at the time of inferential choice. We transduced pyramidal cells of the dorsal hippocampal CA1 (dCA1) with the yellow light-driven neural silencer Archaerhodopsin-T fused with the green fluorescent protein reporter (ArchT-GFP). Optic fibers were subsequently implanted, targeting bilateral dCA1 with light to suppress neuronal spiking during sensory cue presentation (Figures 2E–2H). Suppressing dCA1 spiking impaired inference: light delivery during auditory cues Xn in the inference test (50% of test trials for both set 1 and 2) prevented ArchT-GFP mice from expressing significant reward-seeking bias to Xn cues in set 1 relative to set 2 (Figure 2I). dCA1 light delivery did not impair the reward-seeking bias in GFP control mice (Figure 2I). Furthermore, light delivery during the visual cues Yn, presented after the inference task was complete, did not impair anticipatory reward-seeking behavior of ArchT-GFP mice (Figure 2J). Thus, dCA1 is necessary for inference while dispensable for visual discrimination and first-order conditioning.

### Selective Hippocampal Spiking Response to Task Cues: in Mice

**Figure 3:**
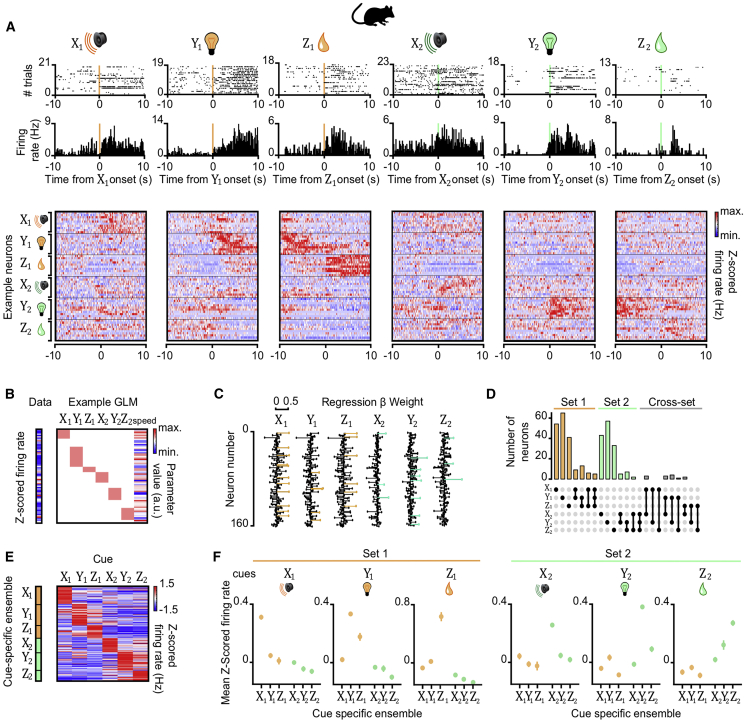
dCA1 Neuronal Representation of Inference Task Cues in Mice (A) Top and middle rows: Raster plots and peri-stimulus time histograms for 6 example neurons, each showing firing response to one of the 6 task cues (Xn, Yn, and Zn) (Figure 1A). Bottom row: heatmap showing the average *Z* scored firing rate (Hz) of 60 example neurons in response to task cues, ordered according to the preferred cue on the y axis. (B) For each recorded neuron, the average *Z* scored firing rate across each trial was filtered by the “decision point” (Figure S4) and regressed onto a GLM that modeled all task cues and the mouse’s average speed. GLM for an example neuron is shown. (C) For each cue, the regression weights from all neuron-specific GLMs are shown for an example recording day. Neurons with regression weights more than 2 SD from the group mean were assigned to a cue-specific ensemble and color-coded for visualization (orange, set 1; green, set 2). (D) UpSet plot (Lex et al., 2014) showing the number of neurons within and shared across each cue-specific ensemble. Only a minority of neurons contributed to more than one cue-specific ensemble. (E and F) Average *Z* scored firing rate of neurons in the Xn,Yn, and Zn ensembles in response to each task cue. (E) For an example recording day, the average response of each neuron allocated to a cue-specific ensemble is shown. (F) Across all recording days, the average response of all neurons in a given cue-specific ensemble is shown (mean ± SEM). See also Table S4.

Using *in vivo* electrophysiology to record dCA1 ensembles in mice, we observed neurons with increased spiking during either the auditory, visual, or outcome cue in both set 1 and set 2 (Figure 3A). To identify neurons showing preferential firing to Xn, Yn, or Zn, we applied a general-linear model (GLM) to spiking activity monitored during each task cue, with the obtained regression weights indicating the response magnitude of each neuron (Figures 3B and 3C). We observed largely non-overlapping neuronal ensembles representing the different task cues (Figures 3D–3F). This suggests dCA1 has the capacity to selectively represent each of the discrete sensory cues and outcomes experienced in the inference task.

### The Hippocampus Computes a Prospective Code during Inference: in Humans and Mice

**Figure 4:**
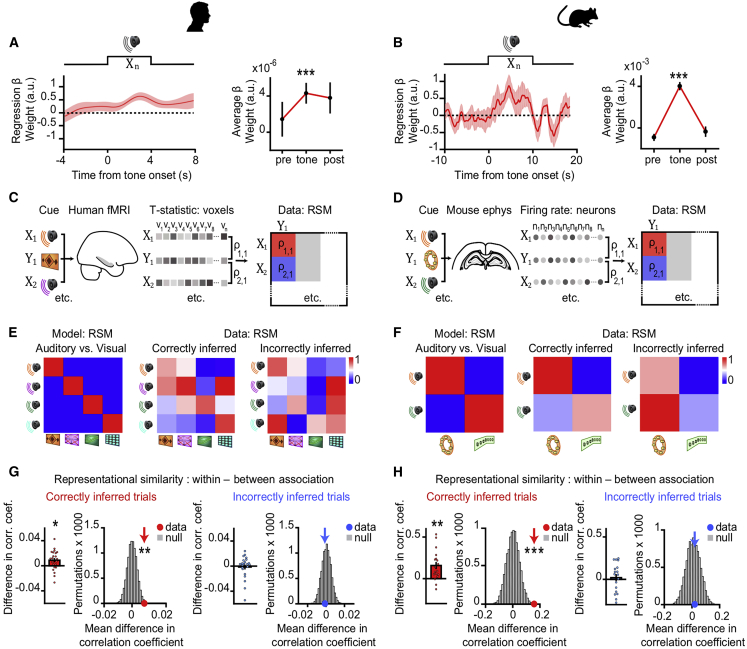
Hippocampal Prospective Memory Code during Correct Inference in Humans and Mice (A and B) During auditory cues Xn in the inference test, the BOLD signal (humans; A) and the *Z* scored firing rate of each dCA1 neuron (mice; B) were regressed onto the behavioral performance (correct versus incorrect inference) using a GLM. During Xn, the extracted regression weights (mean ± SEM) were significantly positive for correct versus incorrect inference (humans: t_21_ = 4.04, p < 0.001; mice: t_99_ = 10.24, p < 0.001). (C and D) In humans (C) and mice (D) hippocampal activity patterns during auditory cues in the inference test and during visual cues in conditioning were compared using RSA to establish a cross-stimulus representational similarity matrix (RSM). In mice, data were filtered by the “decision point” on each trial. (E and F) Average RSMs in humans (E) and mice (F), for the model (predicted results) and the data (observed results), split by behavioral performance in the inference test. Rank-transformed and scaled between (0 to 1) for visualization. (G and H) In both humans (G) and mice (H), hippocampal activity during correctly inferred auditory cues Xn in the inference test significantly predicted the associated visual cue Yn, ([average within association XnYn correlations, RSM main diagonal] – [average between association XnYm correlations, RSM off-diagonals]; correct and incorrect inference; humans: Z_21_ = 2.24, p = 0.013 and Z_21_ = 0.71, p = 0.238; mice: Z_17_ = 3.00, p = 0.001 and Z_17_ = 0.39, p = 0.348; mean ± SEM). In both species, the group mean was compared against a null distribution generated by permuting the identity labels of cues Xn (correct and incorrect inference; humans: p = 0.005 and p = 0.620; mice: p < 0.001 and p = 0.417; alpha set to 0.05). All statistical tests for correctly inferred trials remained significant with Bonferroni correction to account for two comparisons (correctly and incorrectly inferred), alpha set to 0.025. See also Figures S3 and S4 and Table S4.

We asked whether the hippocampus represents the learned and inferred relationships between task cues. First, we assessed evidence for modulation of hippocampal activity during inference in both humans and mice. As reported above, in humans, we observed an increase in the hippocampal BOLD signal during correct versus incorrect inference (Figures 2B and 4A). Similarly, in mice, we observed significant modulation of dCA1 spiking on correct versus incorrect inference trials, after controlling for variance attributed to speed and set (Figure 4B). These findings show modulation of neuronal activity in the mammalian hippocampus during correct inferential choice.

We next investigated the hippocampal computation that serves inference. In the spatial domain, spiking activity in the medial temporal lobe can sweep ahead of an animal’s location (Gupta et al., 2013; Johnson and Redish, 2007; Mehta et al., 2000) and predict subsequent behavior (Ferbinteanu and Shapiro, 2003; Pastalkova et al., 2008; Pfeiffer and Foster, 2013; Singer et al., 2013; Wood et al., 2000). We reasoned that if similar predictive activity serves inferential choice in the cognitive domain, the hippocampus should draw on mnemonic relationships to prospectively represent visual cues Yn in response to auditory cues Xn in the inference test, thereby chaining Xn to outcome Zn.

To test this, we measured hippocampal representations in humans and mice during each auditory cue Xn in the inference test and during each visual cue Yn in the conditioning. We then deployed the same analytical framework across species, applying representational similarity analysis (RSA) (Kriegeskorte et al., 2008; McKenzie et al., 2014; Nili et al., 2014) to both voxels (humans) and neurons (mice) (Figures 4C and 4D). We observed similar results in humans and mice: when the correct outcome was inferred behaviorally, hippocampal activity during the current auditory cue Xn showed higher representational similarity with the associated visual cue Yn, compared to the non-associated (cross-set) visual cues Ym (Figures 4E–4H, S3C, and S3D). This set-selective discrimination in the hippocampal code was not explained by the temporal proximity between inference test trials and re-conditioning trials (Figures S1A and S1B). Therefore, at the time of inferential choice, presentation of Xn elicited representations of the expected Yn cues in a set-specific manner. This suggests hippocampal activity represents learned associations to predict the short-term future, thereby engaging a prospective code to “look ahead” within the current spatial context.

**Figure S4:**
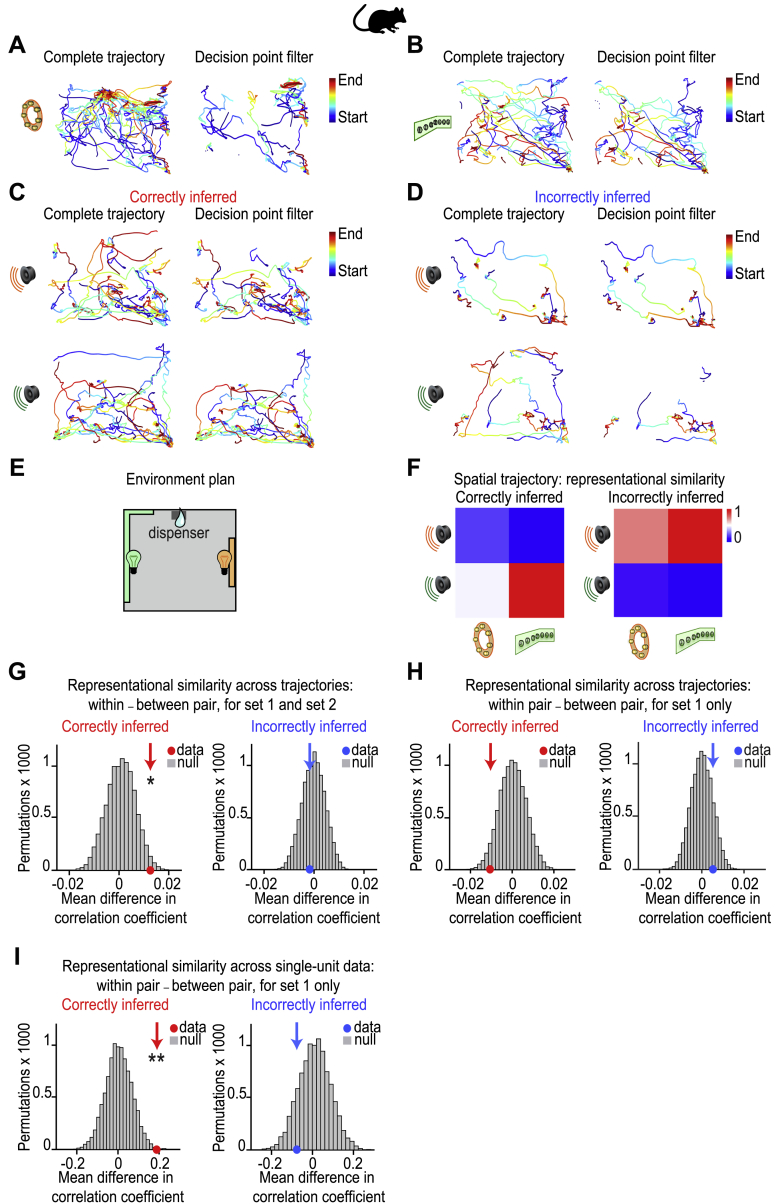
In Mouse dCA1, Representational Similarity between Activity Patterns for Xn and Yn in Set 1 Is Not Explained by Spatial Trajectory, Related to Figures 3, 4, 5, and 6 In mice: (A-D) Overlaid trajectory for an example mouse during visual cues (Yn) and auditory cues (Xn). Blue indicates the start of the trajectory and red indicates the end of the trajectory. Left hand panels: complete trajectories during the cue. Right hand panels: trajectories filtered by the “decision point” of the mouse, as applied in Figures 3, 4, 5, and 6. The “decision point” of the mouse is defined as the time point where the speed of the mouse was below 5cm/s prior to visiting the outcome area on that trial (see STAR Methods). Filtering the trial data by the decision point eliminated time periods where the mouse was at or near the dispenser, thus controlling for spatial confounds in set 1. (A) Trajectories during visual cue Y1 from set 1. (B) Trajectories during visual cue Y2 from set 2. (C) Trajectories during auditory cues Xn from set 1 and 2 for correctly inferred trials. (D) Trajectories during auditory cues Xn from set 1 and 2 for incorrectly inferred trials. (E) Schematic showing birds-eye view of the open field used for the example mouse shown in *A-D*. (F) The average representational similarity matrices (RSMs) across recording days for spatial trajectories during cues Xn and Yn, after filtering by the decision point and dividing the data by performance in the inference test. Rank-transformed and scaled between [0 to 1] for visualization purposes. (G-H) The average representational similarity for ‘within set’ versus ‘between set’ spatial trajectories across cues Xn and Yn, after filtering by the decision point and splitting by performance in the inference test. The group mean was compared against a null distribution generated by permuting the identity labels assigned to the auditory cues Xn. (G) Across both set 1 and 2, spatial trajectories during the auditory cues Xn significantly predicted the trajectories for the associated visual cue Yn, ([within set XnYn correlation] – [between set XnYn correlation], ‘correct inference’ p = 0.014, ‘incorrect inference’ p = 0.687). (H) Across set 1 alone, spatial trajectories during the auditory cues Xn did not significantly predict the trajectories for the associated visual cue Yn, ([within XnYn correlation] – [between XnYn correlation], ‘correct inference’ p = 0.957, ‘incorrect inference’ p = 0.151). (I) Across set 1 alone, the single-unit activity in neurons recorded from dCA1 during auditory cues Xn significantly predicted the activity patterns for the associated visual cue Yn during correct but not incorrect inference ([within set XnYn correlation] – [between set XnYn correlation]). The group mean was compared against a null distribution generated by permuting the identity labels assigned to the auditory cues Xn (‘correct inference’ p = 0.002, ‘incorrect inference’ p = 0.865). Given the absence of significant spatial correlations for set 1 shown in *H*, this result shows that during correct inference, dCA1 ensemble activity predicted the associated cue Yn over and above the spatial location of the animal.

Notably, in mice, these results did not reflect the animal’s location (Figure S4). In humans, where we controlled for value by including multiple visual cues Yn for each outcome Zn (Figure S1D), hippocampal activity during Xn was not explained by the associated value of Yn (Figures S3G–S3I). During inference, the hippocampus therefore appears to draw on memories to forecast the learned consequence of sensory cues (Xn→Yn).

### The Hippocampal Prospective Code Preserves Learned Temporal Statistics: in Mice

**Figure 5:**
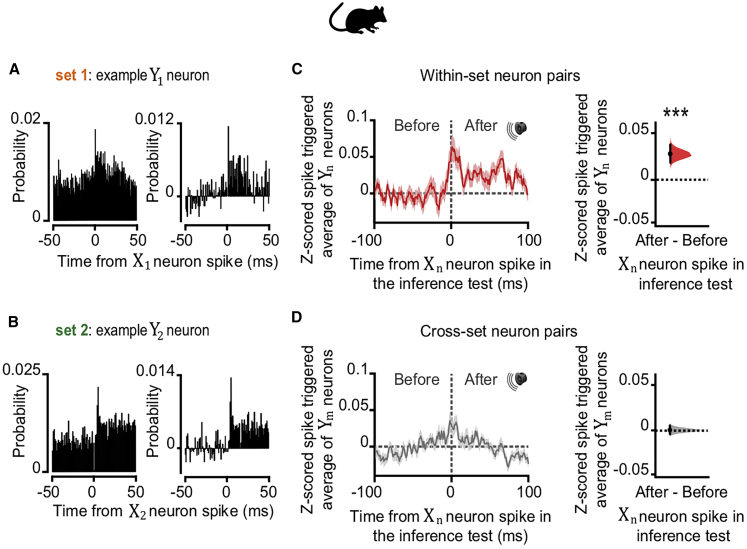
dCA1 Neuronal Spike Timing Supports a Prospective Code during Inference in Mice (A and B) During inference, we assessed the spike time relationships between neuronal ensembles representing cues Xn and Yn (Figures 3C and 3D). The cross-correlogram for spike counts in each pair of XnYn cells showed the spiking probability of neuron Yn relative to neuron Xn. Shown: example cell pair for set 1 (A) and set 2 (B) where neuronal ensembles representing cues Xn and Yn fire sequentially, preserving the learned temporal dynamics of task cues. Right panel: change in joint probability of XnYn spiking relative to baseline (average joint probability 50 ms prior to Xn spikes). (C and D) During cues Xn in the inference test we estimated the *Z* scored spike-triggered average for neurons in ensembles Yn (shown in C) or Ym (shown in D), within a 200-ms window relative to spikes in neurons representing Xn. DABEST plots used to compare the difference in the mean *Z* scored spike-triggered average for both Yn and Ym, “after” minus “before” spikes in Xn: black dot, mean; black ticks, 95% confidence interval; filled-curve, sampling-error distribution. Yn, but not Ym, neurons typically fired after Xn neurons (“after” – “before” Xn spike discharge: red within-set neuronal pairs shown in C, X1Y1 and X2Y2, p < 0.001; gray cross-set neuronal pairs shown in D, X1Y2 and X2Y1, p = 0.983; Figure S5). Thus, despite the absence of Yn cues, spiking across XnYn cell pairs preserved the learned temporal statistics from the observational learning stage. See also Figure S4 and Table S4.

**Figure S5:**
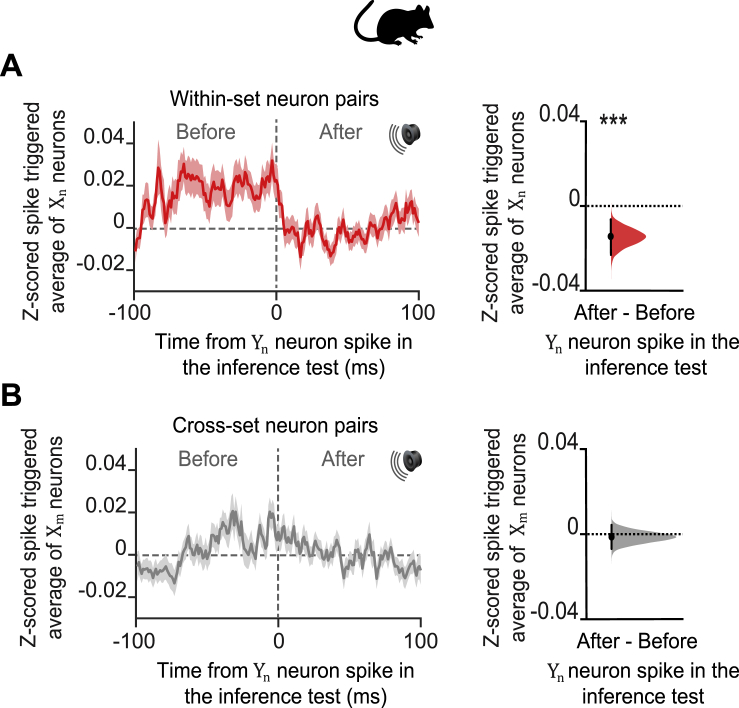
Spike Time Relationships between dCA1 Neurons during Inference, Related to Figure 5 In mice: (A-B) During the auditory cues Xn in the inference test we estimated the Z-scored spike-triggered average for neurons in ensembles Xn, within a 200-ms window relative to the spike times of neurons in ensembles Yn. For each cell pair we assessed the difference in the mean Z-scored spike-triggered average for Xn during the 100-ms interval “after” minus “before” spikes in Yn. The effect size for the difference (“after” – “before”, right-hand panel) was estimated by computing 10,000 bias-corrected bootstrapped resamples (Efron, 2000) and visualized using DABEST plots (Ho et al., 2019): *black-dot*, mean; *black-ticks*, 95% confidence interval; *filled-curve*, sampling-error distribution; *red*, within-set cell pairs; *gray*, cross-set cell pairs. (A) For all within-set neuronal pairs (X1Y1 and X2Y2), the Z-scored spike-triggered average for Xn was significantly higher during the 100 ms before Yn spike discharge (“after” – “before” Yn neuron spike: p < 0.001), showing that during presentation of the auditory cues Xn in the inference test, Xn neurons tend to spike before Yn neurons, thus preserving the temporal statistics of cue presentation from the observational learning stage of the task (Figure 1A). (D) For all cross-set neuronal pairs (X1Y2 and X2Y1), there was no significant temporal bias in the Z-scored spike-triggered average for Xm when comparing the 100 ms “after” minus “before” spikes in Yn (“after” – “before” Xn neuron spike: p = 0.640).

To assess whether this prospective code preserves the statistics inherent to the observational learning, we took advantage of the high temporal resolution of *in vivo* electrophysiology. Taking the neuronal ensembles that selectively represent either Xn or Yn cues (Figures 3C–3F), we assessed the temporal order of spiking activity for pairs of Xn and Yn neurons upon presentation of Xn during the inference test. For within-set XnYn neuronal pairs, neurons representing Yn were significantly more likely to spike after neurons representing Xn (Figures 5 and S5), preserving the temporal relationship between cues Xn and Yn experienced during observational learning despite no further presentation of Yn cues at this stage. Thus, during inference, hippocampal activity represents a prospective code that reflects mnemonic recall for learned temporal statistics.

### The mPFC and Midbrain Represent the Inferred Outcome: in Humans

**Figure 6:**
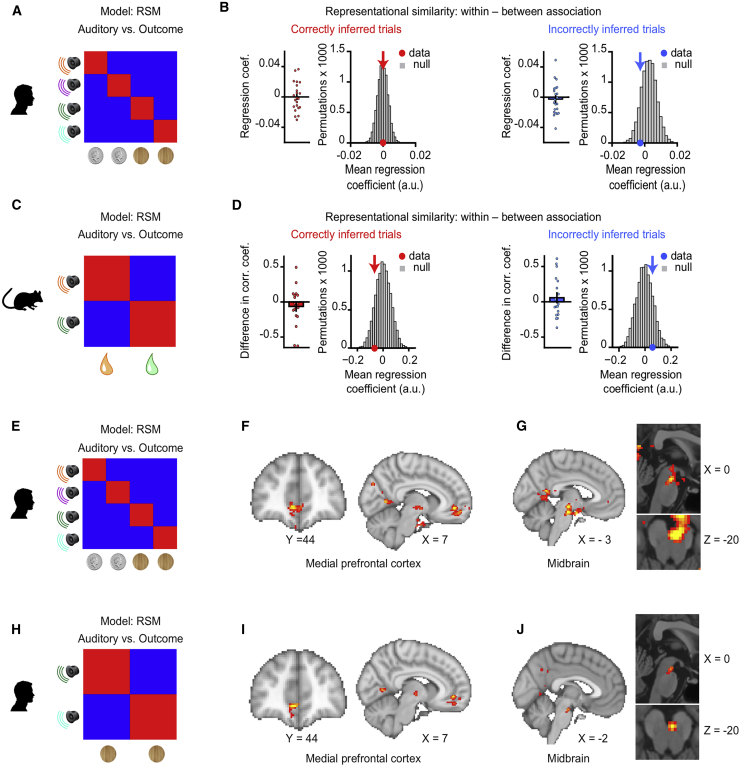
Inferred Outcomes Are Represented in mPFC and Midbrain (A–D) In humans (A and B) and mice (C and D) hippocampal activity patterns during auditory cues Xn in the inference test were compared with activity patterns during outcome Zn in conditioning using RSA. In mice, time bins with SWRs were excluded. Activity patterns during Xn in the inference test, but not Zn in conditioning, were filtered by the “decision point” on each trial, such that representations of Zn were spatially decoupled from representations of Yn. Hippocampal activity in humans and mice did not significantly predict activity associated with the relevant outcome Zn ([average within association XnZn correlations, RSM main diagonal] – [average between association XnZm correlations, RSM off-diagonals]; correct and incorrect inference; humans, conditional on intermediary cues: Z_21_ = −0.06, p = 0.526, and Z_21_ = −0.75, p = 0.772; mice: Z_17_ = −0.87, p = 0.808; and Z_17_ = 0.44, p = 0.331; mean ± SEM), including when comparing the group mean against a null distribution generated by permuting the identity labels for cues Xn (correct and incorrect inference; humans: p = 0.464, and p = 0.868; mice: p = 0.829 and p = 0.176). See Figure S6C for results in humans unconditional on intermediary cues. By contrast, robust dCA1 representation of Zn was observed in mice during experience of outcome Zn in conditioning trials (Figure 3). (E–J) In humans, searchlight RSA with multiple regression (Figures S6D and S6E) was used to identify regions showing representational similarity between auditory cues Xn in the inference test and outcomes Zn in conditioning. During correctly inferred cues Xn, both the mPFC (F) and putative dopaminergic midbrain (G) regions showed significant representation of the associated Zn, conditional on sensory cues that predicted the outcome as shown in the model RSM in (E) (F, mPFC: t_21_ = 5.09, p = 0.003; G, midbrain: t_21_ = 5.56, p < 0.001; thresholded at p < 0.01 uncorrected for visualization purposes only). This result held for cues in set 2 as shown in the model RSM in (H) (I: mPFC: t_21_ = 4.60, p = 0.006; J, midbrain: t_21_ = 3.37, p = 0.027; peak-level FWE corrected using small-volume correction; Figures S6F and S6G; thresholded at p < 0.01 uncorrected for visualization purposes only). See also Figure S4 and Tables S3 and S4.

**Figure S6:**
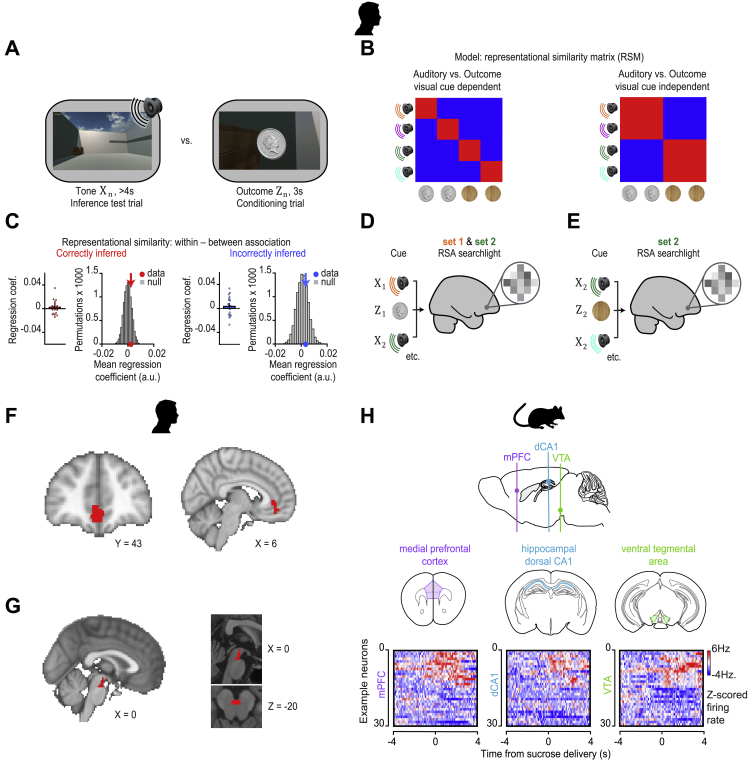
Representation of the Inferred Outcome in Humans and Triple-Site Recording of Neuronal Ensembles in dCA1, mPFC, and VTA in Mice, Related to Figure 6 (A-G) In humans. (A) RSA was assessed between auditory cues Xn during the inference test, and outcome cues Zn during the conditioning trials. (B) Using multiple regression, representational similarity between the auditory cues Xn and outcome cues Zn was assessed using two models. The first model (left) mapped the relationship between the auditory cues Xn and the associated outcomes Zn, conditional on the intermediary visual cues Yn (Figures 6A and 6B). The second model (right) mapped relationships between the auditory cues Xn and the associated outcomes Zn, unconditional on the intermediary visual cues Yn. (C) RSA was applied to the BOLD signal extracted from the hippocampal ROI shown in Figure S3F. Using multiple regression to regress the data onto the two models described in *B*, during the inference test we assessed evidence for representation of outcome cues Zn in the hippocampus. During both correctly inferred and incorrectly inferred trials in the inference test, hippocampal activity did not significantly predict activity associated with the relevant outcome cues Zn, unconditional on intermediary visual cues Yn (correct and incorrect inference: Z_21_ = 0.91, p = 0.182 and Z_21_ = 0.84, p = 0.199). The group mean was further compared against a null distribution generated by permuting the identity labels assigned to the auditory cues Xn (correct and incorrect inference: p = 0.199 and p = 0.289). Results conditional on the intermediary visual cues Yn are shown in Figures 6A and 6B. (D-E) Searchlight RSA in humans was used to identify brain regions showing representational similarity between auditory cues Xn and outcomes Zn for cues in both set 1 and 2 (*D*) and for cues in set 2 only (*E*), where Z2 represents a neutral cue. (F-G) ROIs used to correct for multiple comparisons within the medial prefrontal cortex and VTA (Figures 6I and 6J). (F) An ROI in the medial prefrontal cortex, defined from functional map showing evidence for novel conjunctive representations in medial prefrontal cortex (Barron et al., 2013). (G) An ROI in the VTA, defined from a functional map identifying midbrain activation to reward-prediction error (Klein-Flügge et al., 2011). (H) In mice. Upper: Schematic showing the 3 simultaneously recorded brain regions: medial prefrontal cortex (mPFC), hippocampal dCA1 and ventral tegmental area (VTA) (n = 4 mice). Lower: Heatmap showing spiking activity of 3x30 example neurons recorded across the mPFC, dCA1 and VTA. For each heatmap, each row shows the Z-scored firing rate (Hz) of a given neuron within 100 ms bins, averaged across multiple trials in which sucrose is delivered to the outcome dispenser within the open field shown in Figure 1B. For each brain region the y axis is organized to first show those neurons that show an increase in their spiking activity in response to sucrose delivery. This triple-site recording shows that spiking activity in these three brain regions can be modulated in relation to experience of reward, and also illustrates how triple-site recordings may be used in the future to investigate findings reported in humans at a cellular level (Figure 6).

Having shown that both the human and mouse hippocampi represent the intermediary visual cue Yn in response to the auditory cue Xn during inference (Figures 4 and 5), we asked where in the brain this prospective memory code (Xn → Yn) is translated into a representation of the inferred outcome Zn. We capitalized on the many-to-one mapping between task cues in humans, where representations of Yn and Zn could be dissociated (Figures S1C and S1D). In response to Xn, there was no evidence for representation of the inferred outcome Zn in the human hippocampus (Figures 6A, 6B, and S6A–S6C), with analogous findings in mice (Figures 6C and 6D). This result contrasts with our data recorded in mice during the conditioning stage where dCA1 ensembles show robust responses to the experienced outcome Zn (Figure 3). This suggests that inferential decisions are not supported by a mechanism whereby Xn acquires the value of Yn during encoding (Figures S3G–S3I, 6A, and 6B), or by a mechanism whereby mnemonic information is recirculated within the medial temporal lobe to represent the inferred outcome within the hippocampus (Kumaran, 2012; Kumaran and McClelland, 2012) (Figures 6A, 6B, and S6C). Instead, during inference, the hippocampus uses a prospective code to forecast the learned consequences of sensory cues (Xn→Yn).

To identify where this prospective code is translated into a representation of the inferred outcome, we took advantage of near-whole brain imaging in humans. Using an RSA searchlight to sweep through the entire imaged brain volume (Figures S6D and S6E), we identified regions showing significant representational similarity between auditory cues Xn and inferred outcomes Zn. When the correct outcome was inferred during the inference test, activity patterns in both mPFC and midbrain showed significant representational similarity with the inferred outcome Zn (Figures 6E–6G), even when restricting analyses to neutral cues alone (Figures 6H–6J). Notably, representation of Zn was conditional on the cues that predicted Zn (Figures 6E and S6B), suggesting the inferred outcome is computed online according to a model of the task. Moreover, by representing value-neutral sensory features, the processing specificities of mPFC and putative dopaminergic midbrain regions may go beyond reward or direct reinforcement, consistent with recordings in rodents (Sadacca et al., 2016; Stalnaker et al., 2019; Takahashi et al., 2017). To detail the interaction between mPFC, midbrain, and dCA1 ensembles during inference, further multi-brain-site recordings in animal models will be required, as illustrated during exposure to outcome Z1 (Figure S6H).

### Hippocampal SWRs Nest Mnemonic Short-Cuts for Inferred Relationships: in Mice

Using data acquired during inferential choice in both humans and mice, we showed evidence for a prospective retrieval mechanism that forecasts learned associations, thus indirectly relating cues Xn and Zn (Figures 4, 5, and 6). However, complementary mechanisms may directly link Xn to Zn. One candidate mechanism involves using multiple memories to internally simulate and cache statistics of the environment (Sutton, 1991). In the spatial domain, temporally compressed simulations of previous experience occur in hippocampal SWRs during periods of awake immobility (rest) and sleep (Buzsáki, 2015; Foster, 2017; Joo and Frank, 2018). SWR-related activity could extend beyond direct experience by recombining and recoding mnemonic information (Buzsáki, 2015; Diekelmann and Born, 2010; Lewis and Durrant, 2011; Shohamy and Daw, 2015; Zeithamova et al., 2012b). Accordingly, we tested whether hippocampal SWR activity effectively “autocompletes” the firing associations representing unobserved (yet logical) relationships between cues Xn and Zn. While non-invasive methods can provide a macroscopic index for memory reactivation, accessing the unique electrophysiological profile of SWRs (Buzsáki, 2015) requires invasive methods. The following analyses were therefore restricted to electrophysiological recordings in mice.

**Figure 7:**
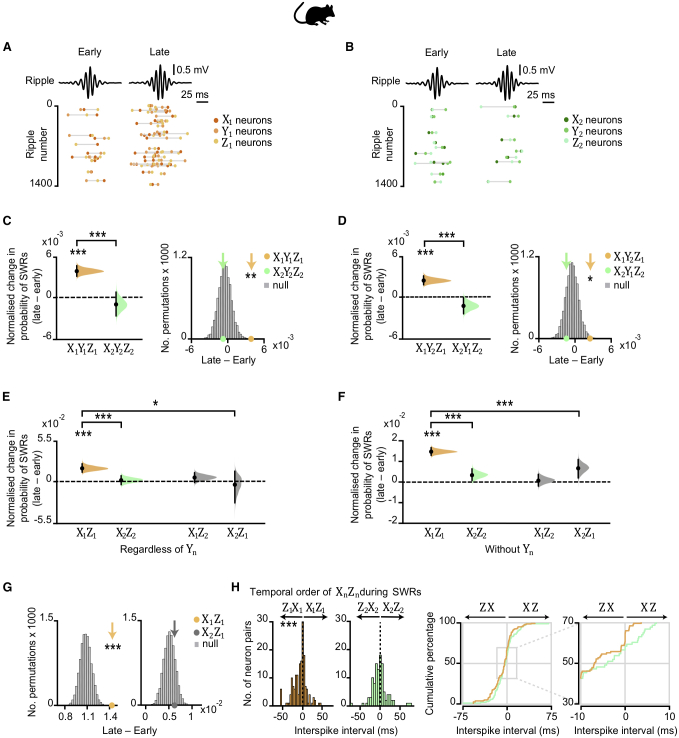
Hippocampal Spiking Represents Inferred Relationships during SWRs (A and B) In mice. Top: mean ripple-band (135–250 Hz) dCA1 oscillations during awake rest in the inference task. Bottom: co-firing of example X1Y1Z1 (A) and X2Y2Z2 (B) neuronal triplets across SWRs during early and late recording days. (C–G) From early to late days (recording days 1–4 versus 5–8; Figure S1B), we estimated the normalized probability of SWRs co-representing neuronal triplets/doublets during awake rest in the inference test. The effect size for the difference between early and late days is shown using a DABEST plot: black dot, mean; black ticks, 95% confidence interval; filled-curve, sampling-error distribution. Tukey’s post hoc multiple comparison test was used to assess the difference in group means following ANOVA. (C) From early to late days, we observed a significant interaction in the probability of SWRs representing set 1 (X1Y1Z1) versus set 2 (X2Y2Z2) neuronal triplets (set × day interaction, two-way ANOVA: F_(1,1573)_ = 28.14, p < 0.001). Tukey’s post hoc test showed a significant increase in SWRs representing set 1 (X1Y1Z1) triplets (p < 0.001). Compared to a null distribution generated by permuting the identity labels of ensemble neurons (Figures 3C and 3D), we again observed a significant increase in SWRs representing set 1 (X1Y1Z1) triplets (p = 0.002). (D) From early to late days we observed a significant interaction in the probability of SWRs representing X1Y2Z1 versus X2Y1Z2 triplets, where the identity of Ym cells were from the opposite set (set × day interaction, two-way ANOVA: F_(1,900)_ = 33.97, p < 0.001). Tukey’s post hoc test showed a significant increase in SWRs representing X1Y2Z1 triplets (p < 0.001). Compared to a null distribution generated by permuting the identity labels of ensemble neurons (Figures 3C and 3D), we again observed a significant increase in SWRs representing X1Y2Z1 (p = 0.014). (E and F) From early to late days we observed a significant interaction in the probability of SWRs representing neuronal pairs for the inferred relationship in set 1 (X1Z1) versus set 2 (X2Z2), both regardless of Yn neurons (E) and in the absence of Yn neurons (F) (set × day interaction, two-way ANOVA: E, F_(1,285)_ = 24.04, p < 0.001; F, F_(1,1716)_ = 45.8, p < 0.001). Tukey’s post hoc test showed a significant increase in SWRs representing set 1 (X1Z1) pairs (E, p < 0.001; F, p < 0.001). This result was not explained by a mere increase in SWRs representing Z1 as the interaction remained significant when comparing SWRs representing set 1 (X1Z1) versus cross-set (X2Z1) pairs (set × day interaction, two-way ANOVA: E, F_(1,260)_ = 5.14, p = 0.024; F, F_(1,1642)_ = 18.08, p < 0.001). (G) To further control for a mere increase in SWRs representing Z1 cues in (F), we compared the group mean against a null distribution generated by permuting the SWRs with Xn spikes, while holding Zn spikes fixed. From early to late days the probability of SWRs showing coactivity between neurons representing X1 and Z1, but not X2 and Z1, increased relative to the respective null distribution (X1Z1: p < 0.001; X2Z1: p = 0.193; Figure S7C). (H) Inter-spike intervals for Xn and Zn neuron pairs. Across all neuron pairs, the percentage of pairs where Z1 fired before X1 was significant (p < 0.001, binomial test with alpha set to 0.05). The percentage of pairs where Z2 fired before X2 did not differ from 50% (p = 0.101, Binomial test with alpha set to 0.05). Set 1 in orange; set 2 in green. The effect for set 1 remained significant with Bonferroni correction for two comparisons (set 1 and set 2) and alpha at 0.025. See also Table S4.

For each recording day (Figures 1D and S1B), we calculated the probability that SWRs nest spikes from neuronal triplets, where each cell provides a (non-overlapping) representation of one of the task cues Xn, Yn, or Zn (Figures 7A and 7B). When comparing early versus late days, the probability that awake SWRs jointly represent all three cues (Xn, Yn, and Zn) significantly increased for set 1 but not for set 2 (Figure 7C). This result suggests that reward-related activity is prioritized in hippocampal SWRs, consistent with work investigating replay of previous experience in SWRs (Singer and Frank, 2009).

**Figure S7:**
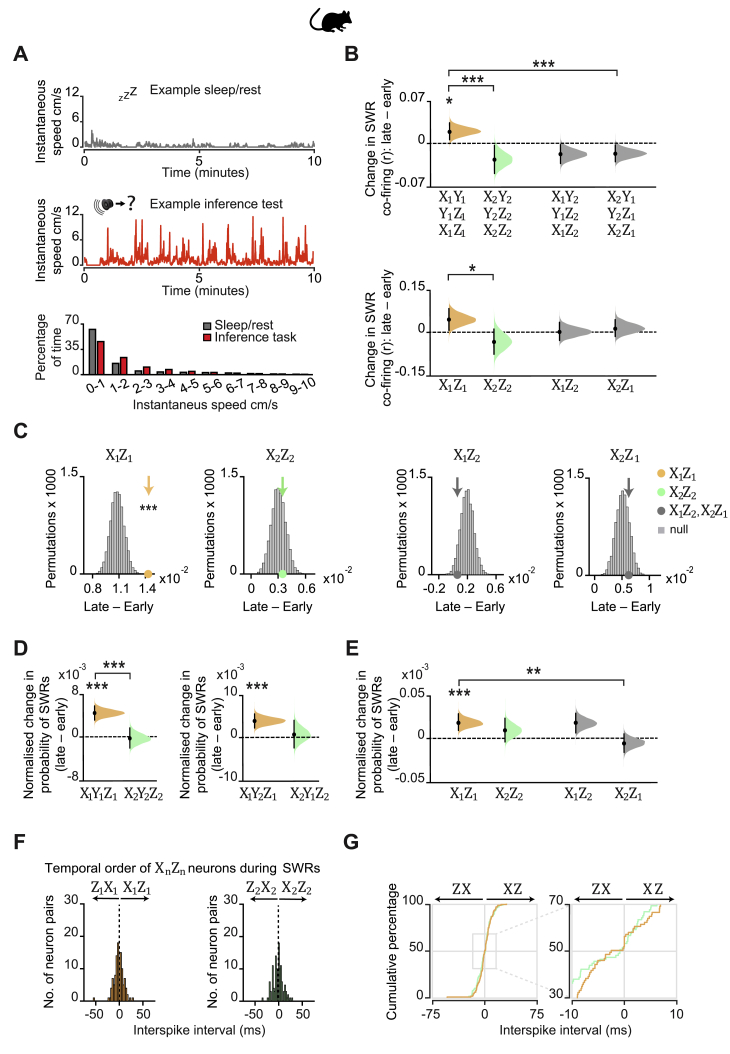
During SWRs, Hippocampal Co-firing Autocompletes Inferred Relationships, Related to Figure 7 In mice: (A) Upper: Example instantaneous speed (cm/s) during a rest/sleep session of a recording day (Figure S1B) when the mouse was in the “sleep box”. Middle: Example instantaneous speed (cm/s) during an inference test session (Figure S1B) when the mouse was in the open field (Figure 1B). Notably, the mouse is more active during the inference test relative to the rest/sleep session shown in *A*. Lower: The distribution of instantaneous speed (mean ± SEM) recorded across all mice during rest sessions and during the inference task. The proportion of time spent immobile (0-1cm/s) was greater during rest sessions, while the proportion of time spent active (> 1cm/s) was greater during the inference task. (B) To further measure firing associations between hippocampal neurons during SWRs (Figure 7), we used a second approach reported previously (Dupret et al., 2010; McNamara et al., 2014). In brief, this co-firing measure involved first calculating for each cell the instantaneous firing rate counts within each SWR of a given recording session, before computing the Pearson correlation coefficient between the instantaneous firing rate counts for each cell pair, across all SWRs of that recording session. Between early and late recording days, we then tested the difference in pairwise co-firing for cue-defined cell pairs against zero and estimated DABEST plots as described in Figures 1E and 1F. Tukey’s post hoc multiple comparison test was used to further assess the difference in group means following ANOVA. Upper: From early to late recording days we observed a significant interaction in SWR co-firing for set 1 versus set 2 cell pairs (set x day interaction, two-way ANOVA: F_(1,2005)_ = 13.68, p < 0.001). Tukey’s post hoc test revealed a significant increase in SWR co-firing for set 1 cell pairs ([X1Y1, Y1Z1, X1Z1]: p = 0.040) and a significant decrease in set 2 cell pairs ([X2Y2, Y2Z2, X2Z2]: p = 0.040). This result was not explained by a mere increase in the probability of SWRs representing Z1: the interaction remained significant when comparing the SWR co-firing for set 1 and equivalent across-set ([X2Y1, Y2Z1, X2Z1]) cell pairs (two-way ANOVA: F_(1,1965)_ = 13.93, p < 0.001), where Tukey’s post hoc test also revealed a significant increase in SWR co-firing for set 1 cell pairs ([X1Y1, Y1Z1, X1Z1]: p = 0.015). Lower: From early to late recording days we observed a significant interaction in SWR co-firing for set 1 versus set 2 cell pairs representing the inferred (but not directly observed) relationship (set 1: [X1Z1]; set 2: [X2Z2]; set x day interaction, two-way ANOVA: F_(1,365)_ = 6.45, p = 0.012). However, Tukey’s post hoc test did not reveal a significant increase in SWR co-firing for set 1 cell pairs ([X1Z1]: p = 0.235). Critically, unlike the analyses presented in Figure 7, the methodological approach implemented here did not control for SWR co-firing of cells representing the intermediary visual cues Yn. (C) From early to late recording days we observed a significant increase in the probability of SWRs representing neuronal pairs for the inferred relationship in set 1 (X1Z1) in the absence of Yn neurons (Figure 7F). To control for a mere change in SWRs representing task cues from early to late recording days, we compared the group mean for both within-set (X1Z1 and X2Z2) and cross-set (X1Z2 and X2Z1) neuronal pairs against a null distribution generated by permuting the SWRs in which Xn neuronal spikes occurred in each pair, while holding the relevant Zn neuronal spikes fixed and thus preserving the average firing rate of both Xn and Zn cells. This analysis revealed a significant increase in probability of SWRs representing the inferred relationship from set 1 (X1Z1, p < 0.001), but no significant change in the probability of SWRs representing all other neuronal pairs (X2Z2, p = 0.281; X1Z2, p = 0.934; X2Z1, p = 0.193). See also Figure 7G. (D-E) From early to late recording days, the normalized probability of SWRs co-representing neuronal triplets during periods of rest/sleep sessions recorded in the “sleep box” at the beginning and end of the recording day (Figure S1B). The effect size for the difference between early and late is shown using DABEST plots as described in Figures 1E and 1F. Tukey’s post hoc multiple comparison test was used to further assess the difference in group means following ANOVA. (D) From early to late recording days we observed a significant interaction in SWRs representing set 1 (X1Y1Z1) versus set 2 (X2Y2Z2) neuronal triplets (set x day interaction, two-way ANOVA: F_(1,1106)_ = 15.86, p < 0.001). Tukey’s post hoc test revealed a significant increase in the probability of SWRs representing set 1 (X1Y1Z1) neuronal triplets (p < 0.001). From early to late recording days there was no significant interaction in the probability of SWRs representing set 1 (X1Y2Z1) versus set 2 (X2Y1Z2) neuronal triplets, where the identify of Yn cells were from the opposite set (set x day interaction, two-way ANOVA: F_(1,828)_ = 2.67, p = 0.102). However, Tukey’s post hoc test revealed a significant increase in the probability of SWRs representing set 1 (X1Y2Z1) neuronal triplets (p = 0.024). (E) From early to late recording days there was no significant interaction in the probability of SWRs representing neuronal pairs for the inferred relationship in set 1 (X1Z1) versus set 2 (X2Z2), regardless of Yn neurons (set x day interaction, two-way ANOVA: F_(1,224)_ = 1.41, p = 0.237). However, Tukey’s post hoc test revealed a significant increase in SWRs representing set 1 (X1Z1) neuronal pairs (p = 0.005). This result was not explained by a mere increase in SWRs representing Z1 as a significant interaction was observed when comparing SWRs representing set 1 (X1Z1) versus across-set (X2Z1) neuronal pairs (set x day interaction, two-way ANOVA: F_(1,187)_ = 10.94, p = 0.001). Overall, during offline periods of rest/sleep the increase in SWRs co-representing X1Z1 neuronal pairs showed less specificity than that observed for SWRs recorded in periods of quiet wakefulness during the inference test (Figures 7C–7F). (F-G) Inter-spike intervals for Xn and Zn neuron pairs (see STAR Methods). Unlike SWRs recorded during awake rest in the inference test (Figure 7H), during periods of sleep/rest in the “sleep box” the number of neuron pairs where Z1 fired before X1 was not significant (p = 0.195, Binomial test). Similarly, the number of neuron pairs where Z2 fired before X2 did not differ from 50% (p = 0.519, Binomial test). Set 1 in orange; set 2 in green.

This result did not merely reflect simulation of an internal model of the inference task (X1→ Y1→ Z1), because the probability that SWRs co-represent X1 and Z1 together with the visual cue from the alternative set, Y2, similarly increased (Figure 7D). Indeed, regardless of the intermediary visual cue Yn, the probability that SWRs co-represent the auditory cue X1 together with the logically associated outcome Z1 increased with behavioral experience of the task (Figures 7E and S7B). Furthermore, with the recorded ensemble of neurons at hand, the probability that a given awake SWR represents the inferred relationship (X1, Z1) in the absence of the intermediary cue (Y1) also increased with experience (Figure 7F). These results suggest SWRs represent a mnemonic short-cut for inferred relationships that include reward.

These findings cannot be explained by a mere increase in SWRs representing reward (Z1) as the observed increase in representation of set 1 cell pairs, X1Z1, was significantly greater than equivalent changes in the cross-set cell pairs, X2Z1 (Figures 7E and 7F). Moreover, unlike cross-set cell pairs, the increase in probability that a given awake SWR represents co-activity for the inferred relationship (X1, Z1) occurred over and above any change in activity for cells representing either X1 or Z1 cues (Figures 7G and S7C). Comparable results were observed during offline periods of sleep but with lower fidelity (Figures S1B, S7D, and S7E), as reported for replay of spatial firing patterns during awake rest versus sleep (Karlsson and Frank, 2009). Together, these findings suggest that the hippocampal representation of profitable (rewarding) yet unobserved relationships increases in SWRs, thus supporting a direct mnemonic short-cut for inferred relationships.

During awake SWRs, we further assessed the spike time relationships between Xn and Zn cells. Past studies investigating representation of spatial trajectories in hippocampal SWRs report evidence for replay in a temporally reversed order (Csicsvari et al., 2007; Diba and Buzsáki, 2007; Foster and Wilson, 2006; Gupta et al., 2010). Despite cues Xn and Zn never being directly experienced together, here we found that cells representing Z1 fired significantly earlier than cells representing X1 (Figure 7H). Cell pairs representing cues from neutral set 2 (X2 and Z2) showed no such temporal bias (Figure 7H). Thus, cell pairs that included reward representation (X1 and Z1) exhibited reverse firing (Z1→ X1) relative to the inferred direction (X1→ Z1). Consistent with evidence suggesting that hippocampal replay coordinates reward responsive neurons with the dopaminergic midbrain during quiet wakefulness but not sleep (Gomperts et al., 2015), we did not observe offline reverse firing of the inferred relationship (Figures S7F and S7G). This suggests that waking memories may serve reverse sequential firing in awake SWRs to assign credit or allow updates to environmental cues that are logically linked but not directly experienced with reward.

## Discussion

Here, we use a cross-species approach to uncover how the mammalian brain computes inference, a cognitive operation central to adaptive behavior. Across a multi-day inference task, we reveal a cellular-level description of the underlying computation, alongside a macroscopic readout of this process.

Our study shows that during inference, the hippocampus engages a prospective code that preserves the learned temporal statistics of the task. In addition, during rest/sleep in mice, hippocampal SWRs show increased coactivation of neurons representing inferred relationships that include reward. Thus, during rest/sleep the hippocampus appears to “join-the-dots” between discrete items that may be profitable. We propose this mechanism provides a means to build a cognitive map that stretches beyond direct experience, creating new knowledge to facilitate future decisions.

This process of “joining-the-dots” between logically related events is consistent with evidence that SWR spiking is not only determined by prior experience (Buzsáki, 2015; Foster, 2017; Joo and Frank, 2018). Rather, the intrinsic connectivity of the hippocampus (Somogyi and Klausberger, 2017), self-generated sequences (Dragoi and Tonegawa, 2011), forward planning (Ólafsdóttir et al., 2015; Pfeiffer and Foster, 2013), structural knowledge (Liu et al., 2019), and stitching together of spatial trajectories (Gupta et al., 2010; Wu and Foster, 2014) all play a role. Here, hippocampal SWR spiking represents a non-spatial, second-order mnemonic link between items not experienced together, over and above simulating an internal model that draws on direct experience. The reported increase in SWRs nesting inferred relationships suggests hippocampal spiking activity during SWRs may build higher-order relationships to integrate knowledge into a coherent schema (Tse et al., 2007). This new understanding of hippocampal SWRs may explain why sleep/rest facilitates behavioral readouts of insight and inferential reasoning in humans (Coutanche et al., 2013; Ellenbogen et al., 2007; van Kesteren et al., 2010; Lau et al., 2011; Wagner et al., 2004; Werchan and Gómez, 2013).

Consistent with studies showing that reward-related activity influences the spatial content of SWRs (Pfeiffer and Foster, 2013; Singer and Frank, 2009), our findings suggest SWR content can be skewed toward events that are more salient, have greater future utility, and/or generate larger reward-prediction errors. The spiking content reported here may further be prioritized toward active inferential choice, because correct inference in response to X1, but not X2, requires mice to elicit a purposeful action toward the dispenser. While distinct memories encoded close in time are represented by overlapping ensembles (Cai et al., 2016), here, we controlled for this by presenting cues from set 1 and 2 in a randomly interleaved manner during the reconditioning and inference test, thus matching the temporal proximity of within- and between-set cues.

Changes in neuronal coactivation in hippocampal SWRs are suitable to influence wide-spread cortical and subcortical targets, directly or via intermediate relay regions (Battaglia et al., 2011; Buzsáki, 2015; Joo and Frank, 2018). This may explain how the putative dopaminergic midbrain acquires a representation of the inferred outcome (Zn) in response to a preconditioned cue (Xn), which cannot be accounted for by temporal difference learning algorithms (Sadacca et al., 2016). Specifically, hippocampal SWR spiking may broadcast value information to relate reward information received at the end of a sequence to earlier events (Foster et al., 2000; Sutton, 1988). Consistent with this hypothesis, reverse replay in awake SWRs occurs during reward-motivated spatial behavior (Diba and Buzsáki, 2007; Foster and Wilson, 2006; Gupta et al., 2010), while our data show an inverted temporal order in non-spatial inferred relationships. SWR-nested spiking may therefore facilitate retrospective credit assignment or value updating of sensory cues represented by the mPFC and midbrain, even if those cues are not directly paired with an outcome. Such cross-region coordination may explain why functional coupling observed between hippocampus and mPFC during post-encoding rest predicts measures of memory integration in humans (Schlichting and Preston, 2016). In this manner, SWR-related hippocampal training signals may alleviate the computational cost of inference by building a model or “cognitive map” of the external world that spans multiple brain regions.

In addition to this SWR-related mechanism during rest/sleep, we show in mice that dCA1 pyramidal neurons are necessary for inferential choice. Moreover, during inference in both humans and mice, the hippocampus represents a veridical copy of learned associations in temporal sequence (Xn→Yn). These findings were not explained by mere spatial location, yet these temporally structured mnemonic associations may be analogous to spatial sequences of place cells (e.g., McNaughton et al., 1983; Mehta et al., 2000). Sequential firing of this kind may be a necessary requirement for a brain region evolved to support memory (Buzsáki and Moser, 2013).

Previous studies suggest that during learning, memories for past overlapping events can be evoked and associated with newly encountered information to link memories across experiences (Nagode and Pardo, 2002; Schlichting et al., 2014; Shohamy and Wagner, 2008; Zeithamova and Preston, 2010; Zeithamova et al., 2012a). This integrative encoding may even assign value to stimuli not directly paired with an outcome (Wimmer and Shohamy, 2012), alleviating the need to recall intermediary cues at the time of choice. However, previous studies have not differentiated between representation of the intermediary (Yn) and inferred cues (Zn) during inferential choice, leaving the underlying mechanism ambiguous. Here, in humans, we dissociate representations of the intermediary (Yn) and inferred cues (Zn) by using a many-to-one mapping between cues. At the time of choice, this paradigm shows evidence for hippocampal representation of the intermediary cue (Yn), but not the inferred outcome (Zn) or value associated with Yn. We also show that mouse hippocampal dCA1 is necessary for inference. Together, these results suggest inferential choice is supported by a hippocampal mechanism where mnemonic sequences are recalled “on-the-fly.” This mechanism may further depend upon extra-hippocampal regions representing the relevant sensory cues. Thus, while our findings are not contradictory to previous human fMRI studies, by dissociating representations of Yn from Zn at the time of inference, we propose the hippocampus draws on learned experience, while other downstream circuits may use the hippocampal output to reinstate an integrated or overlapping neural code.

Using near-whole brain imaging in humans, we show that during inferential choice the inferred outcome (Zn) is represented in mPFC and midbrain, even when the corresponding outcome is neutral. This highlights a division of mnemonic labor between the hippocampus on the one hand, and the mPFC and (putative dopaminergic) midbrain on the other: whereas the hippocampus draws on learned sequences (Xn→Yn), the hypothetical inferred outcome (Zn), rewarding or neutral, is represented in the mPFC and midbrain, potentially inherited by integrative encoding or spiking activity during SWRs. Inference therefore involves a memory recall mechanism that spans multiple brain regions. This differs from computational models that propose associative information is integrated locally within the medial temporal lobe via recurrent loops (Kumaran, 2012), but is consistent with evidence showing representation of intermediary cues in the medial temporal lobe at the time of choice (Koster et al., 2018). Moreover, our findings support evidence suggesting the mPFC uses an abstract model of the environment to guide behavior (Hampton et al., 2006), while the midbrain supports learning of relationships that extend beyond those associated with direct reinforcement (Langdon et al., 2018; Sadacca et al., 2016; Sharpe et al., 2017; Stalnaker et al., 2019; Takahashi et al., 2017). Retaining both veridical mnemonic recall and allowing inference for higher-order relationships provides the comprehensive cognitive flexibility necessary for adaptive mammalian behavior in an ever-changing environment.

The inference task was implemented across multiple days and may therefore generalize to everyday examples of inference where individuals draw upon information learned across days, weeks, or even years. While training demands in rodents made this multi-day paradigm inevitable, we note that our results could, in part, reflect the consequence of this schedule. For example, by conducting each task stage on a separate day, we mitigated against the formation of overlapping neuronal codes for distinct memories encoded close in time. Our task design also lends toward using more mature or consolidated memories: evidence in rodents suggests memories are rapidly generated in both hippocampus and mPFC, gradually becoming quiescent in hippocampus with consolidation in mPFC (Kitamura et al., 2017; Preston and Eichenbaum, 2013; Squire et al., 2015). If training demands allowed the inference task to be performed within 1 day, the inferred outcome may be represented in both the hippocampus and mPFC, rather than mPFC and midbrain, as observed here. Notably, our paradigm differs from several studies investigating inferential reasoning in humans within one day (Koster et al., 2018; Preston et al., 2004; Schlichting et al., 2014; Wimmer and Shohamy, 2012; Zeithamova et al., 2012a). Unveiling the precise temporal dependency of the computation supporting inference will require further work.

Recording and manipulating neural dynamics will help establish an understanding of the mechanisms underlying adaptive and maladaptive behavior (Deisseroth, 2014). However, cellular recordings and causal manipulations are normally performed using invasive methods in animal models where it is difficult to translate the identified mechanisms into an understanding of human behavior. In attempt to overcome this explanatory gap, here we use a cross-species approach to take advantage of complementary tools available in humans and mice. Despite differences between the mouse and the human brain, a cross-species approach remains justified by the preserved homology of mammalian neural circuits. However, there are inevitable limitations associated with comparing data across species. When investigating aspects of higher-order cognition, perhaps the greatest limitation resides in our inability to verbally communicate with animals. While humans received explicit instructions and comprehension was monitored throughout, mice had to reveal task rules via trial and error, with no social obligation to cooperate. Despite keeping the experimental paradigm the same across mice, their behavior was more variable. The difference in our ability to instruct/train humans and mice also led to differences in task design, where humans were able to learn a many-to-one mapping between cues to permit dissociation of neuronal representations. Nevertheless, by implementing a comparable task in humans and mice, and acquiring data in an iterative manner, we show how results from one species can guide the course of investigation in the other. We propose this cross-species approach provides a foundation for innovative multidisciplinary investigation of brain functions, in both physiological and pathophysiological conditions.

In summary, our study reveals the functional anatomy and neuronal computation underlying inferential reasoning in the mammalian brain. We implement a cross-species approach in humans and mice to integrate data from whole-brain imaging, cellular-level electrophysiology, and optogenetic manipulations of the same behavior. In doing so, we reveal a holistic description of the neural computation underlying inference in the mammalian brain. We identify a critical role for the hippocampus, which engages a prospective memory code during inferential choice and represents a cognitive short-cut for inferred relationships that include reward in rest/sleep. By unveiling these neuronal mechanisms, we show how the brain can generate new knowledge beyond direct experience, thus supporting high-level cognition.

## STAR★Methods

### Key Resources Table

**Table:** 

REAGENT or RESOURCE	SOURCE	IDENTIFIER
**Bacterial and Virus Strains**		

rAAV2-CAG-flex-ArchT-GFP	UNC Vector Core	N/A
rAAV2-CamKII-ArchT-GFP	UNC Vector Core	N/A
rAAV2-CAG-flex-GFP	UNC Vector Core	N/A
rAAV2-CamKII-GFP	UNC Vector Core	N/A

**Experimental Models: Organisms/Strains**		

CaMKII-Cre mice	The Jackson Laboratory	https://www.jax.org Stock #005359; RRID: IMSR_JAX:005359
C57BL/6J mice	Charles River, UK	https://www.criver.com/Strain code: 632

**Software and Algorithms**		

MATLAB	Mathworks	https://www.mathworks.com Version: 2016b
Psychtoolbox-3	Psychtoolbox developers	http://psychtoolbox.org Version: 3.0.13
SPM	FIL Methods group, University College London (UCL)	https://www.fil.ion.ucl.ac.uk/spm Version: SPM 12
RSA Toolbox	Nili et al., 2014	http://www.mrc-cbu.cam.ac.uk/methods-and-resources/toolboxes/
Unity	Unity Technologies, CA United States	https://unity.com/Version: 5.5.4
Intan RHD2000	Intan Technologies, Los Angeles	http://intantech.com/products_RHD2000.html Version: RHD2164
KlustaKwik	K. Harris	https://github.com/klusta-team/klustakwik/
Kilosort via SpikeForest	Magland et al., 2020; Pachitariu et al., 2016	https://github.com/cortex-lab/KiloSort via SpikeForest https://github.com/flatironinstitute/spikeforest
Imetronic POLYtrack	Imetronic	http://www.imetronic.com/

**Other**		

MR compatible headphones	Sensimetrics	https://www.sens.com/Type: S14 inset earphones
7 Tesla Magnetom MRI scanner	Siemens	N/A
1-channel transmit and a 32-channel phased-array head coil	Nova Medical, USA	N/A
Arduino microcontroller development board	Arduino online store	https://store.arduino.cc/usa/Product: Arduino Mega 2560 Rev3
Adafruit Motor/Stepper/Server Shield for Arduino v2	Adafruit online store	https://www.adafruit.com/Product: Adafruit Motorshield V2
12um tungsten wires	California Fine Wire	https://calfinewire.com/Product: M294520
Optic fibers	Doric lenses, Québec, Canada	http://doriclenses.com/Product: MFC_200/230-0.37_10mm_RM3_FLT
Head-stage amplifier	Intan Technologies, Los Angeles	https://www.intantech.com Product: RHD2164
561nm diode-pumped solid-state laser	Laser 2000, Ringstead, UK	Product: CL561-100-0

### Resource Availability

#### Lead Contact

Further information and requests for resources and reagents should be directed to and will be fulfilled by the Lead Contact, David Dupret (david.dupret@bndu.ox.ac.uk).

#### Materials Availability

This study did not generate new unique reagents.

#### Data and Code Availability

The data and code used in this study will be made available via the MRC BNDU Data Sharing Platform (https://data.mrc.ox.ac.uk/) upon reasonable request.

### Experimental Model and Subject Details

#### Mouse subjects

24 mice were included in the study (age of 4-6 months, 24 males) (Table S4). Mice were heterozygous for the transgene expressing the Cre recombinase under the control of the CaMKIIa promoter and maintained on a C57BL/6J background (Jackson Laboratories; CamIIa-Cre B6.Cg-Tg(Camk2a-cre)T29-1Stl/J, stock number 005359, RRID: IMSR_JAX:005359) or were wild-types with a C57BL/6J background (Charles River, UK). Animals had free access to water in a dedicated housing facility with a 12/12 h light/dark cycle (lights on at 07:00h). Animals were housed with their littermates up until the start of the experiment, during which they were housed alone. Food was available *ad libitum* before the experiments (see below). All experiments involving mice were conducted according to the UK Animals (Scientific Procedures) Act 1986 under personal and project licenses issued by the Home Office following ethical review.

#### Human subjects

22 healthy human volunteers participated in the study (mean age of 22.8 ± 0.74 years, 4 males). All experiments involving humans were approved by the University of Oxford ethics committee (reference number R43594/RE001). All participants gave informed written consent.

### Method Details

#### Mouse inference task environment

During both the pre-training and the inference test protocols (see below), mice were allowed to explore a square-walled open-field enclosure (46 cm width, 38 cm high-walls) within which they were presented with a range of different sensory stimuli controlled via a single-board microcontroller (Arduino Mega 2560 Rev3). Two speakers placed above the open-field were used to deliver the auditory cues (Xn) which constituted pure tones (frequency: 10KHz and 2KHz). Two LED panels were affixed to walls of the open-field which when illuminated served as visual cues (Yn): an L-shaped set of green LEDs with main strip spanning the width of one wall, and a circular set of orange LEDs affixed to a different wall (Figure 1B). A liquid dispenser/aspirator fitted with an infra-red beam detecting lick events was used to deliver/remove the outcome (Zn) which constituted either a drop of 15% sucrose solution (reward; set 1) or a drop of water (neutral; set 2). The outcome cues (Zn) were only available for 10 s before being automatically aspirated by the dispenser.

#### Human inference task environment

The virtual reality (VR) environment simulating the open-field enclosure used for mice was coded using Unity 5.5.4f1 software (Unity Technologies, CA United States) and included a square-walled room with no roof. To help evoke the experience of 3D space and aid orientation within the VR environment, each wall of the environment was distinguished by color (dark green, light green, dark gray or light gray), illumination (two walls were illuminated while the other two were in shadow) and by the presence of permanent visual cues. The permanent visual cues included clouds in the sky, a vertical black stripe in the middle of the light green wall, a horizontal black strip across the light gray wall, and a wooden box situated in one corner of the environment. A first-person perspective was implemented and participants could control their movement through the virtual space using the keyboard arrows (2D translational motion) and the mouse-pad (head tilt). Movement through the environment elicited the sound of footsteps.

Within the VR environment participants were exposed to a range of different sensory stimuli. Auditory cues (Xn) constituted 80 different complex sounds (for example, natural sounds or those produced by musical instruments) that were played over headphones. Four different visual cues (Yn) could appear on the walls of the environment, each of which had a unique color and pattern. Two of the visual cues were always presented on the same wall, the assignment of which was randomized for each participant. The two remaining visual cues were ‘nomadic’, meaning that with each presentation they were randomly assigned to one of the four walls. A wooden box situated in the corner of the environment served to deliver the outcome cues (Zn) which constituted either a rewarding silver coin or a neutral wood-chip. To harvest the value of a silver coin (20 pence) (reward) or woodchip (0 pence) (neutral), participants were required to first collide with the wooden box which caused the wooden walls to disappear, and second collide with the coin or wood-chip which was accompanied by a ‘collision’ sound. The outcome cues (Zn) were only available for 10 s. The cumulative total value of harvested reward was displayed in the upper left corner of the computer screen.

#### Humans and mice: inference task overview

In the respective environments described above, both humans and mice performed an *inference task*. The task was adapted from associative inference and sensory preconditioning tasks described elsewhere (Brogden, 1939; Preston et al., 2004; Robinson et al., 2014) and involved 3 stages (Figures 1A, S1A, and S1B). First, in the ‘observational learning’ stage, subjects learned a set of associations between auditory and visual cues via mere exposure. On each trial, an auditory and visual cue (Xn and Yn) were presented serially and contiguously: auditory cue (mice: 10 s, humans: 8 s) followed by associated visual cue (mice: 8 s; humans: 8 s). Second, in the ‘conditioning’ stage, subjects learned that half the visual cues predicted delivery of a rewarding outcome (‘set 1’), while the other half predicted delivery of a neutral outcome (‘set 2’). On each trial a visual cue and outcome (Yn and Zn) were presented serially and contiguously: visual cue (mice: 8 s; humans: 8 s) followed by outcome delivery (mice: 10 s available from liquid dispenser; humans: 6 s available in wooden box). Finally, in the ‘inference test’ stage subjects were exposed to the auditory cues (Xn) in isolation. In response to each auditory cue, subjects could infer the appropriate outcome using the learned structure of the task. This test thus provided an opportunity to investigate inferential choice. In both species the 3 stages of the task were performed across at least 3 consecutive days (see below).

To match task difficulty and avoid ceiling effects in human task performance, in the observational learning stage we scaled the number of associative memories learned by human subjects relative to mice. Consequently, between auditory and visual cues there was a one-to-one mapping in mice and a many-to-one mapping in humans (Figure S1C). In addition, in mice we included one visual cue *per* set, while in humans we included two visual cues *per* set (Figure S1D). Therefore, in total mice learned two auditory-visual associations in the observational learning stage and two visual-outcome associations in the conditioning stage, while humans learned eighty auditory-visual associations in the observational learning stage and four visual-outcome associations in the conditioning stage. At the start of the experiment the pairings between auditory, visual and outcome cues were randomly assigned for each human participant and for each mouse.

#### Mouse pre-training protocol

Mice were preselected by assessing their propensity to lick drops of sucrose solution delivered at a liquid dispenser when food restricted to 90% their free-feeding body weight. Selected mice were then fed *ad libitum* up until day 4 of the pre-training.

During the pre-training, mice first completed the observational learning stage, conducted across 6 consecutive days (Figures S1B and S1F). Each day the mice were placed in the open-field environment for 20 separate sessions, each lasting ∼8-10 minutes. Each session included 6 trials where an auditory cue (Xn) was followed by presentation of the associated visual cue (Yn), from either set 1 or 2. The inter-trial interval (ITI) was ∼1.5 minutes. On day 1, each session included cues from either set 1 or set 2 (‘blocked’), while on days 2-6, sessions could include cues from either set 1 or 2 (‘blocked’), or both set 1 and 2 presented in a pseudo-random order (‘mixed’). On each day of training, cues from set 1 and 2 were presented equally often. Trials were triggered only when the animal was moving. To prepare for the next stage of the task (conditioning), across the final 3 days of the observational learning stage mice were food restricted to reach 90% their free-feeding body weight.

After the observational learning stage, the conditioning was conducted across 4-5 consecutive days (Figures S1B and S1F). Each day the mice were placed in the open-field environment for 20 separate sessions, each lasting ∼8-10 minutes. Each session included 6 trials where a visual cue (Yn) was presented followed by the associated outcome (Zn), a drop of sucrose for set 1, or a drop of water for set 2. The ITI was ∼1.5 minutes. On day 1 of the conditioning stage, each session included cues from either set 1 or set 2 (‘blocked’), while on day 2-4/5 of the conditioning stage, each session included cues from either set 1 or 2 (‘blocked’), or from both set 1 and 2, presented in a pseudo-random order (‘mixed’). Trials were triggered only when the animal was moving. The animal’s propensity to visit the dispenser was assessed and the number of cues presented from set 1 and 2 adjusted accordingly. Thus, mice that were prone to approach the dispenser received additional set 2 trials, while mice that were less inclined to approach the dispenser received additional set 1 trials. Across mice, the ratio of set 1 to set 2 trials delivered during conditioning did not predict subsequent performance on the inference test (r_22_ = −0.06, p = 0.816).

#### Human pre-training protocol

On the first day of the experiment participants performed the *observational learning* during which participants were required to learn at least 40 (out of 80 total) auditory-visual associations (Figures S1C and S1E). This training occurred within the VR environment and was divided into 8 sub-sessions. In each sub-session, participants controlled their movement within the VR environment and were presented with 20 trials in which 10 different auditory-visual associations were each presented twice, in a random order. The ITI was 5 s. Participants were given the choice to repeat the sub-session if they so wished. After the sub-session, learning of auditory-visual associations was monitored outside the VR environment, using an observational learning test coded in MATLAB 2016b using Psychtoolbox (version 3.0.13). On each trial of the observational learning test, 1 auditory cue (Xn) from the sub-session was presented, followed by presentation of 4 different visual cues. Participants were instructed to select the visual cue (Yn) associated with the auditory cue (Xn), using a button press response within 3 s and were only given feedback on their average performance at the end of the test. Each auditory cue in the sub-session was presented 2 times. Participants were required to repeat this training in the VR environment (including the observational learning test) until they obtained at least 50% accuracy for auditory-visual associations in the sub-session. In total, 3 participants were unable to reach this learning criteria of 50% accuracy and were excluded after the first training day.

After obtaining at least 50% accuracy on the observational learning test for each sub-session, participants were given a master test. The master test had the same format as the observational learning test, except that it included all 80 auditory cues, each of which was presented 3 times. Training on the observational learning stage was terminated when participants reached 50% accuracy on the master test. If participants failed to reach 50% accuracy, training in the VR environment was repeated for those sub-sessions with poor performance.

On the second day of the experiment participants underwent the conditioning, where they learned that 2 of the 4 visual cues were associated with a virtual silver coin (later converted to a monetary reward of 20p per coin) on 80% of trials. The remaining 2 visual cues were associated with a virtual wood-chip, a neutral outcome of no value (0p per chip) on 100% of trials. Training occurred within the VR environment and on each trial, participants were presented with a visual cue (Yn) followed by delivery of the rewarding monetary coin or neutral wood-chip (Zn) to a wooden box. Participants were instructed to only look in the wooden box after the visual cue was presented and instructed to leave the wooden box before the next trial. The inter-trial interval ITI was 2 s.

Performance during the VR conditioning training was monitored using a conditioning test coded in MATLAB 2016b using Psychtoolbox (version 3.0.13). On each trial of the conditioning test, participants were presented with a still image of a visual cue before being asked to indicate the probability of reward using a number line. Participants were given 3 s to respond and were only given feedback on their average performance at the end of the test. Participants were required to repeat the VR conditioning training and conditioning test until they performed the test with 100% accuracy (Figure S1E).

On day 3 of the experiment, before entering the MRI scanner, participants repeated the conditioning test. Participants then entered the 7T MRI scanner and performed the fMRI scan task (see below and Figure S1A). Immediately after exiting the scanner, participants were given a surprise observational learning test, equivalent to the master test performed on day 1.

#### Mouse inference test protocol

After mice completed the training protocol (observational learning and conditioning stages) we implemented an inference test on up to 8 consecutive recording days (Figure S1B). On each test day we first reconditioned mice to show a reward-seeking bias to visual cues (Yn) in set 1 relative to those in set 2, across two consecutive sessions of at least 12 trials presented in a pseudo-random order. During this reconditioning, reward-seeking behavior was quantified as time spent in the outcome area during the visual cues (Yn), prior to outcome delivery (Figure 1D), for set 1 (Y1) minus set 2 (Y2) (i.e., difference from zero). All mice included in the analysis exhibited a reward-seeking bias during the reconditioning, as defined by requiring fewer reconditioning sessions to show a bias than 2 standard deviations from the group average. In total, two mice were excluded from the study as their number of trials required to achieve reward-seeking bias during reconditioning exceeded 2 standard deviations from the mean.

Mice then proceeded to the inference test where auditory cues (Xn) were presented in isolation for a total of 10 s, followed by an ITI of at least 30 s. Auditory cues (Xn) from set 1 and set 2 were presented in a pseudo-random order, with 26 trials per day. During the inference test, reward-seeking behavior was quantified as the time spent in the outcome area in the 20 s period after the offset of the auditory cues (Figure 1D). The reward-seeking bias was quantified as the difference in reward-seeking behavior for set 1 and set 2 against zero: X1- X2. After each block of inference test trials (8-10 trials), mice were removed from the open-field to rest, before being given a brief block of reconditioning trials (Figure S1B). This interleaved paradigm (reconditioning-test-reconditioning-test etc.) was designed to minimize extinction effects in response to the auditory cues. Finally, at the end of each test day, mice were re-exposed to the observational learning stage (Figures 1A and S1B).

The location of mice implanted with tetrodes was tracked using 3 LED clusters attached to the Intan board (see below) connected to the microdrive (McNamara et al., 2014); mice implanted with optic fibers only were tracked by contrast against the floor of the open-field (Imetronic, France). For both approaches the location of the animal was captured at 25 frames per second using an overhead camera.

#### Human inference test protocol

The inference test was incorporated into the fMRI scan task which included two different trial types: inference test trials and reconditioning trials (Figures 1C and S1A). For both types of trials, participants viewed a short video taken from the VR training environment. The videos were presented via a computer monitor and projected onto a screen inside the scanner bore. On each trial the duration of the video was determined using a truncated gamma distribution with mean of 7 s, minimum of 4 s and maximum of 14 s. During reconditioning trials, the video of the VR environment orientated toward a visual stimulus displayed on one of the four walls. At the end of the video, participants were presented with a still image of the associated outcome for that visual cue (Figure 1C). During the inference test trials, the video of the VR environment was accompanied by an auditory cue, played over MR compatible headphones (S14 inset earphones, Sensimetrics). Visual cues were not displayed during these trials. At the end of the video, participants were presented with a question asking ‘Would you like to look in the box?’, with the options ‘yes’ or ‘no’ (Figure 1C). Participants were required to make a response within 3 s using an MR compatible button box and their right index or middle fingers. No feedback was given. To control for potential confounding effects of space, each video involved a trajectory constrained to a 1/16 quadrant of the VR environment, evenly distributed across the different visual and auditory cues. Across conditioning trials, each visual cue was presented 16 times, once in each possible spatial quadrant. Across inference test trials, each of the 80 possible auditory cues was presented once, and for each set of auditory cues Xn (determined by the associated visual cues Yn) the spatial quadrant of the accompanying videos were evenly distributed across all quadrants. The fMRI scan task was evenly divided across 2 scan blocks, each of which lasted 15 minutes.

#### Mouse surgical procedures

All surgical procedures were performed under deep anesthesia using isoflurane (0.5%–2%) and oxygen (2 l/min), with analgesia provided before (0.1 mg/kg vetergesic) and after (5 mg/kg metacam) surgery.

For optogenetic manipulations (n = 15 mice), viral injections were targeted bilaterally to dCA1 using stereotaxic coordinates (−1.7mm and −2.3mm anteroposterior from bregma, ± 1.7 mm lateral from bregma, and −1.1 mm ventral from the brain surface; 400 nL per site). The adeno-associated viral (AAV) vectors were delivered at a rate of 100 nl/min using a glass micropipette, lowered to the target site and held in place for 4 mins after virus delivery before being withdrawn (McNamara et al., 2014). rAAV2-CAG-FLEX-ArchT-GFP was used for the Cre-dependent expression of ArchT-GFP under CAG promoter in the dCA1 pyramidal neurons of CamKII-Cre mice and rAAV2-CamKII-ArchT-GFP was used for the expression of ArchT-GFP under CamKII promoter in the dCA1 neurons of C57BL/6J mice. For the control experiments we used corresponding AAVs encoding GFP only: rAAV2-CAG-FLEX-GFP in CamKII-Cre mice or rAAV2-CamKII-GFP in C57BL/6J mice. All viruses are from E.S. Boyden (available at UNC Vector Core).

For electrophysiological recordings (n = 10 mice), mice were implanted with a microdrive containing 12-14 independently movable tetrodes that either all targeted the pyramidal layer of bilateral CA1 in the hippocampus (n = 6) (van de Ven et al., 2016) or three brain regions: the pyramidal layer of hippocampal CA1, the medial prefrontal cortex and the ventral tegmental area (triple-site dCA1-mPFC-VTA recordings; Figure S6H). The distance between neighboring tetrodes inserted in a given brain region of each hemisphere was 0.4 mm. Tetrodes were constructed by twisting together four insulated tungsten wires (12 μm diameter, California Fine Wire) which were briefly heated to bind them together into a single bundle. Each tetrode was attached to a 6 mm long M1.0 screw to enable independent manipulation of depth. The microdrive was implanted under stereotaxic control with reference to bregma. For hippocampal dCA1, tetrodes were implanted by first identifying central coordinates −2.0 mm anteroposterior from bregma, ± 1.7 mm lateral from bregma as references to position each individual tetrode contained in the microdrive, and initially implanting tetrodes above the pyramidal layer (∼-1 mm ventral from brain surface). A similar approach was used for tetrodes aimed at the medial prefrontal cortex using central coordinates +1.7 mm anteroposterior from bregma, ± 0.3 mm lateral from bregma, and an initial −1.5 mm ventral from brain surface; and at the ventral tegmental area using central coordinates −3.2 anteroposterior from bregma, ± 0.5 mm lateral from bregma, and an initial −3.8 mm ventral from brain surface. Following the implantation, the exposed parts of the tetrodes were covered with paraffin wax, after which the drive was secured to the skull using dental cement. For extra stability, four stainless-steel anchor screws were inserted into the skull before the drive was implanted. Two of the anchor screws, inserted above the cerebellum, were attached to 50 μm tungsten wires (California Fine Wire) and further served as a ground and reference electrodes during the recordings.

For optogenetic manipulations, optic fibers (230 μm diameter, Doric Lenses, Canada) were incorporated into a microdrive designed to bilaterally deliver light to the dCA1 pyramidal cell layer using stereotaxic coordinates (−2.0 mm anteroposterior from bregma, ± 1.7 mm lateral from bregma, and −1.1 mm ventral from the brain surface). Implantation occurred 2 weeks after dCA1 viral injections.

#### Mouse *in vivo* light delivery

Optical dCA1 stimulation was performed in both ArchT-transduced mice (to optogenetically silence pyramidal neurons) and GFP-transduced control mice using a diode-pumped solid-state laser (Laser 2000, Ringstead) that delivers yellow light (561nm; ∼5-7 mW output power) to the optic fibers implanted above the dCA1 pyramidal cell layer. Using synchronous transistor-transistor logic (TTL) pulses, light was delivered to dCA1 simultaneous with the presentation of either the auditory cues in the inference test (10 s duration; Figure 2I), or with presentation of visual cues presented after both training and the inference test were complete (8 s duration; Figure 2J). Notably, optogenetic suppression of dCA1 neuronal spiking during presentation of these additional visual cues could not affect learning or performance on the inference test.

#### Mouse *in vivo* multichannel data acquisition

During the training protocol mice were gradually accustomed to being connected to the recording system. On the morning of each recording day, single-unit spiking activity together with the electrophysiological profile of the local field potentials (LFPs) were used to adjust the position of each tetrode relative to either the dCA1 pyramidal cell layer, mPFC or VTA. Tetrodes were then left in position for ∼1.5-2 h before recordings started. At the end of each recording day, tetrodes were gently raised by ∼500 μm to avoid possible mechanical damage to their target structure overnight.

Multichannel ensemble recordings were conducted during the inference test protocol. The signals from the electrodes were amplified, multiplexed and digitized using a single integrated circuit located on the head of the animal (RHD2164, Intan Technologies; gain x1000) (McNamara et al., 2014). The amplified and filtered (0.09Hz to 7.60kHz) electrophysiological signals were digitized at 20 kHz and saved to disk along with the synchronization signals from the animal’s position tracking, the presentation of each type of sensory cue, the licks events, and the laser activation. To track the location of the animal, three LED clusters were attached to the headstage and captured at 25 frames per second by an overhead color camera.

#### Mouse spike detection and unit isolation

The electrophysiological data was subsequently bandpass filtered (800Hz to 5kHz) and single extracellular discharges detected through thresholding the root-mean square (RMS) power spectrum using a 0.2 ms sliding window. Detected spikes of the individual electrodes were combined per tetrode. To isolate spikes putatively belonging to the same neuron, spike waveforms were first up-sampled to 40 kHz and aligned to their maximum trough. Principal component analysis was applied to these waveforms ± 0.5 ms from the trough to extract the first 3–4 principal components per channel, such that each individual spike was represented by 12 waveform parameters. For all main analyses, an automatic clustering program (KlustaKwik, http://klusta-team.github.io) was run on the principal component space and the resulting clusters were manually recombined and further isolated based on cloud shape in the principal component space, cross-channel spike waveforms, auto-correlation histograms and cross-correlation histograms (Harris et al., 2000; van de Ven et al., 2016). All sessions recorded on the same day were concatenated and clustered together. Clusters were only included for further analysis if they showed stable cross-channel spike waveforms across the entire recording day, a clear refractory period in the auto-correlation histogram, and well-defined cluster boundaries. For a small subset of our data (Figures 2G, 2H, and S6H), we applied an automated clustering pipeline using Kilosort (https://github.com/cortex-lab/KiloSort) via the SpikeForest sorting framework (https://github.com/flatironinstitute/spikeforest) (Magland et al., 2020; Pachitariu et al., 2016). To apply KiloSort to data acquired using tetrodes, the algorithm restricts templates to channels within a given tetrode bundle, while effectively masking all other recording channels. The resulting clusters were manually curated to check all clusters and remove spurious cells using metrics derived from the waveforms and spike times, and then verified by the operator. This procedure was cross-validated using several datasets and verified against manual curation, by computing confusion matrices to validate that clusters obtained automatically were also obtained with the previous method. In total, 1586 neurons were included in the analyses.

#### Human fMRI data acquisition

The fMRI scan task was performed inside a 7 Tesla Magnetom MRI scanner (Siemens) using a 1-channel transmit and a 32-channel phased-array head coil (Nova Medical, USA) at the Wellcome Centre for Integrative Neuroimaging Centre (University of Oxford). To acquire fMRI data a multiband echo planar imaging (EPI) sequence was used to acquire 50 1.5 mm thick transverse slices with no interslice gap and resulting isotropic voxels of 1.5 × 1.5 × 1.5 mm^3^ resolution, repetition time (TR) = 1.512 s, echo time (TE) = 20 ms, flip angle = 85°, field of view 192 mm, and acceleration factor of 2. To increase SNR in brain regions for which we had prior hypotheses, we restricted the fMRI sequence to a partial volume, thus increasing the number of measurements acquired. The partial volume covered occipital and temporal cortices. For each participant, a T1-weighted structural image was acquired to correct for geometric distortions and perform co-registration between EPIs, consisting of 176 0.7 mm axial slices, in-plane resolution of 0.7 × 0.7 mm^2^, TR = 2.2 s, TE = 2.96 ms, and field of view = 224 mm. A field map with dual echo-time images was also acquired (TE1 = 4.08 ms, TE2 = 5.1 ms, whole-brain coverage, voxel size 2 × 2 × 2 mm^3^).

### Quantification and Statistical Analysis

#### Mouse electrophysiology analysis: inference task

To identify ensembles of neurons representing the six different cues included in the task (Xn, Yn, Zn), we first filtered the data by the “decision point” of the mouse. The “decision point” of the mouse was defined as the latest time bin in the trial of interest where the speed of the mouse was below 5cm/s prior to visiting the outcome area (Figure S4). By excluding data acquired in time bins occurring after the “decision point” of the mouse, we eliminated epochs when the mouse was located at, or approaching, the liquid dispenser. In this manner, we controlled for the spatial location of the mice on each trial.

After filtering the data by the “decision point”, we then visualized the firing response of different dCA1 neurons to each of the task cues (Xn, Yn, Zn; Figure 3A). The instantaneous spike discharge of each neuron was assessed within time bins that spanned a ± 10 s window from onset of each cue. For the peristimulus time histograms, the time bin for estimating the firing rate (Hz) for each neuron was 150 ms. For the heatmap, the time bin for estimating the average Z-scored firing rate for each neuron was 100 ms (Figure 3A).

Across all recorded neurons, within each trial we filtered the data by the “decision point” before estimating the Z-scored firing rate of each neuron during each 100 ms time bin spanning each trial. For each neuron, the Z-scored firing rate across time bins was then averaged for each trial, and the responses across all trials stacked and regressed onto a GLM indicating the identity of the sensory cues presented on each trial (Figure 3B). To control for differences in running speed across trials, we included a dummy variable in the model, indicating the standardized average speed per trial, again filtered by the “decision point” (Figure 3B). For each neuron, this analysis provided a regression weight indicating the extent to which the firing rate of the neuron in question changed in response to each sensory cue. To identify ensembles of neurons representing a given task cue, we selected neurons with a positive beta weight above 2 standard deviations from the mean regression coefficient, calculated across all recorded neurons for that cue (Figures 3C and 3D).

To assess whether successful inferential choice was associated with modulation of hippocampal dCA1 spiking activity, for each recorded neuron we estimated the average Z-scored spike discharge in each 100 ms time bin spanning a 30 s period peristimulus to presentation of each auditory cue Xn in the inference test. For each 100ms time bin, we then regressed the firing rate vector onto behavioral performance (‘1’ for correct inference and ‘0’ for incorrect inference), while accounting for both the speed of the mouse and the set of the auditory cues (set 1 or 2) across trials. Each general linear model (GLM) thus yielded a regression weight reflecting the difference in Z-scored firing rate for correct versus incorrect trials, for a given neuron. For each temporal bin, we then estimated the average regression weight across all recorded neurons, indicating the extent to which dCA1 spiking activity was modulated by behavioral performance (correct versus incorrect inference) through time (Figure 4B).

To assess the representational similarity of dCA1 ensemble firing patterns in response to the six cues included in the inference task, we first established the average Z-scored firing rate of each neuron in 100 ms time bins spanning all trials. For each cue, we then averaged the response of each neuron, before stacking the response across neurons to generate a population vector (Figure 4D). For each task cue, separate population vectors were generated for correct and incorrect trials. A representational similarity matrix (RSM) was then generated for both correct and incorrect trials, using the Pearson correlation coefficient obtained by correlating the population vector for each cue with the population vector for all other cues (Figure 4F). To estimate the representational similarity between auditory and visual cues on each recording day, the average between-association correlation coefficient (RSM off-diagonals: X1 versus Y2, and X1 versus Y2) was subtracted from the average within-association correlation coefficient (RSM main-diagonal: X1 versus Y1, and X2 versus Y2) (Figure 4H). Summary statistics were tested at the group level using two approaches: (1) a one-sided Wilcoxon signed-rank test across recording days; (2) a one-sided permutation test where the null distribution was generated by estimating the group average 10,000 times, after permuting the identity of all auditory cues in the RSM on each iteration. Correct and incorrect trials were kept separate for this permutation test. For visualization of the group average RSM (Figure 4F), the average correlation coefficient was estimated for each auditory-visual pair for each recording day to give a 4x4 matrix.

During presentation of auditory cues Xn in the inference test we used a spike-triggered average to assess the temporal relationship in spiking discharge between pairs of neurons representing Xn and Yn cues (Figures 5C and 5D). Taking ensembles of neurons representing Xn and Yn cues (as defined in Figures 3C and 3D), a 200 ms window was defined around each Xn spike during the within-set auditory cue. For each pair of Xn and Yn neurons, spike counts for each Yn neuron (Yn, within set: Figure 5C; Ym, cross-set: Figure 5D) were summed within each 1 ms bin of the 200 ms window, before estimating the Z-scored average firing rate for each 1 ms bin across all possible Xn spikes in the pair. Those pairs of Xn and Yn neurons where the Yn neurons fired less than 20 spikes across all Xn spikes were excluded from the analysis. For visualization purposes only (Figures 5C and 5D), a moving average was applied to the spike-triggered average, using bin size of 5 ms. An equivalent analysis was performed to assess spike counts in Xn neurons in response to spikes in Yn neurons (Figure S5).

#### Mouse electrophysiology analysis: rest/sleep

SWR events were detected as described previously (McNamara et al., 2014; van de Ven et al., 2016). The LFP signal from the tetrode with the highest number of recorded dCA1 pyramidal neurons was band-pass filtered (135-250 Hz), and the signal from a ripple-free reference tetrode was subtracted to eliminate common-mode noise (such as muscle artifacts). Next the power (root mean square) of the processed signal was calculated. SWR detection was applied to periods of immobility (instantaneous speed below 1.5cm/s), and the threshold for SWR event detection set to 7 standard deviations above the background mean power.

To determine whether triplets of neurons were coactive during SWRs, we estimated the joint-firing probability during SWRs recorded during periods of quiet wakefulness in the inference test (Figure 7) or during periods of long immobility in the sleep/rest session (Figures S7A and S7D–S7G). To control for differences in firing rate across triplets, the joint-firing probability was normalized by the average firing rate of the triplet. Across recording days, we computed the difference in joint-firing probabilities during SWRs that occurred early (recording days 1:4) and late (recording days 5:8) in the inference test (Figure S1B). To assess joint-firing of neurons across Xn, Yn and Zn ensembles (as defined in Figures 3C and 3D), we estimated all possible triplets, and then computed the coactivation probability as follows:

$${\hat{p}}_{early}=\left(n_{early}/N_{early}\right)/f_{early}\;and\;{\hat{p}}_{late}=\left(n_{late}/N_{late}\right)/f_{late}$$

where n_early_ (n_late_) is the number of SWRs during the inference test (Figure 7) or during the rest session (Figures S7D and S7E) during early (late) recording days in which all neurons in the triplet were active; N_early_ (N_late_) is the total number of SWRs during the inference test (Figure 7) or during the rest session (Figures S7D and S7E) during early (late) recording days; f_early_ (f_late_) is the average of the mean firing rate of neurons in the triplet during early (late) recording days. We then tested whether the difference in these probabilities pˆ_diff_ = pˆ_late_ - pˆ_early_, was consistently different from zero, estimating the effect size for the difference by computing 10,000 bootstrapped resamples. Triplets of neurons that were not coactive in any SWRs were not included in the analysis. To assess evidence for increased representation of a cognitive short-cut in SWRs (Xn, Zn), the above analysis was applied to either douplets of neurons regardless of neurons representing Yn (Figure 7E), or to triplets of neurons where the joint-firing of neurons Xn and Zn was considered only in absence of spiking activity in neurons representing Yn (Figures 7F and 7G). To estimate the inter-spike interval between pairs of neurons (Figure 7H, S7F, and S7G), we took only the first spike in the ripple for each neuron, before taking the difference in spike time across both neurons in the pair (i.e., Zn - Xn).

To further illustrate coactivation of neuronal pairs during SWRs (Figure S7B), we used a second approach reported previously (McNamara et al., 2014). In brief, this involved first estimating the instantaneous firing rate counts within each SWRs, before calculating the correlation coefficient between the instantaneous firing rate counts for each cell pair. Between early and late recording days, we tested the difference in correlation coefficients between cell pairs against zero. To estimate the effect size for the difference we computed 10,000 bootstrapped resamples. Notably, this approach did not allow for analysis of triplets, nor permit control for spiking activity in neurons representing Yn.

#### Human fMRI preprocessing and GLMs

Pre-processing of MRI data was carried out using SPM12 (https://www.fil.ion.ucl.ac.uk/spm/). Images were realigned to the first volume, corrected for distortion using field maps, normalized to a standard EPI template and smoothed using an 8-mm full-width at half maximum Gaussian kernel. To remove low frequency noise from the pre-processed data, a high-pass filter was applied to the data using SPM12′s default settings. For each participant and for each scanning block, the resulting fMRI data was analyzed in an event-related manner using four different GLMs, one designed for univariate analyses, a second designed for assessing functional connectivity using a psychophysiological interaction (PPI), and a third and fourth designed for multivariate analyses. All GLMs were applied to data from both scan task blocks. In addition to the explanatory variables (EVs) of interest (described below), in all GLMs 6 additional scan-to-scan motion parameters produced during realignment were included as nuisance regressors to account for motion-related artifacts in each task block.

The first GLM, used to analyze univariate BOLD effects (Figures 2B and 2C), included 14 EVs per block. Of the 14 EVs, 8 accounted for trials in the inference test, divided according to performance of the subject (correct or incorrect inference), before being further divided according to the 4 possible auditory-visual associations. An additional 4 explanatory variables accounted for conditioning trials, divided by the 4 different visual cues. The onset of events within these first 12 EVs were locked to the onset of the video presented in each trial. The 2 final EVs accounted for the onset of questions presented during inference test trials, and the onset of outcomes presented during conditioning trials. To decorrelate the first 12 EVs from the final 2, the duration of onsets for the first 12 EVs was set using a box-car function to 4 s, the minimum duration of the video, whereas the duration of onsets for the final 2 EVs was set to the duration of the outcome/question. All EVs were then convolved with the hemodynamic response function.

The second GLM, used to assess functional connectivity using a PPI (Figure 2D), included 3 EVs per block, describing physiological, psychological and PPI regressors. The physiological regressor was defined from the fMRI time-course extracted from a seed region in the auditory cortex (see ROI definition below). The psychological regressor contrasted trials with correct versus incorrect inference during the inference test. The PPI regressor was constructed by extracting and deconvolving the time-course from the auditory cortex, multiplying it by the psychological regressor and then convolving the output with the hemodynamic response function (HRF). To account for additional unwanted variance, task relevant EVs included in the first GLM described above were also included.

The third GLM, used to assess representational similarity between auditory and visual cues (Figures 4 and S3C–S3I), included a unique EV for each trial included in EVs 1-12 from the first GLM. To maximize cross-voxel sensitivity in the BOLD response to different cues, each unique EV was described by a delta function locked to the end of the video, 4 s after video onset to ensure adequate decoupling from the response to the question or outcome. 2 additional EVs were included to account for the onset of all questions (inference test trials) and the onset of all outcome presentations (conditioning trials), modeled in the same way as in the first GLM. The delta function for each EV was then convolved with the hemodynamic response function.

The fourth GLM, used to assess representational similarity between auditory and outcome cues (Figures 6 and S6A–S6G), included a unique EV for each trial included in EVs 1-8 from the first GLM, and each trial included in the EV accounting for the onset of all outcomes. All auditory cue unique EVs were modeled in the same way as in the third GLM. All outcome cue unique EVs were described by a delta function locked to the onset of the outcome presentation. 2 additional EVs were included to account for the onset of all questions (inference test trials) and the onset of all visual cues (conditioning trials), modeled in the same way as in the first GLM. The delta function for each EV was then convolved with the hemodynamic response function.

#### Human univariate fMRI analysis

Using the output of the first GLM for univariate analysis, the following contrasts were assessed. First, to measure the univariate BOLD response to all inference test trials, the fMRI BOLD signal during inference test trials (EVs 1-8) was contrasted against the fMRI BOLD signal during conditioning trials (EVs 9-12) (Figure 2C). Second, to measure the univariate BOLD response to correct versus incorrect inference, inference test trials where participants made the correct inference (EVs 1-4) were contrasted against those where participants made the incorrect inference (EVs 5-8) (Figure 2B). This second contrast was also used to define the psychological regressor implemented in the PPI described above (Figure 2D). The resulting contrast images for all participants were entered into a second-level random effects ‘group’ analysis.

To visualize the time-course of the hippocampal response to inference (Figure 4A), we extracted the BOLD time series from the preprocessed data of each participant using the hippocampal ROI (Figure S3A). The obtained signal was resampled with a resolution of 300ms, divided into trials on the inference test, and at each time bin the signal was regressed against an EV indicating which trials the participant made the correct versus incorrect inference while accounting for the delay in the haemodynamic response. For each participant, the resulting regression weights were then estimated at each time bin and the average across all participants displayed (Figure 4A).

#### Human multivariate fMRI analysis

The output of the third and fourth GLMs were used to estimate the representational similarity in the BOLD response to different trials, using the representational similarity analysis toolbox (RSA) (Nili et al., 2014). For each trial, a t-statistic map for the relevant EV was estimated (comparing the response to that trial against the baseline).

Using the output t-statistic maps from the third and fourth GLM, activity patterns were extracted from a hippocampal ROI (Figure S3F) and the relative similarity between the response patterns elicited in different trials were assessed using Pearson correlations, and expressed as a correlation coefficient (r). For each participant, the response patterns from trials during the inference test were compared with the response patterns from trials during the conditioning phase, before being represented in a trial-by-trial cross-stimulus representational similarity matrix (RSM) [response to inference test by response to conditioning trials] (Figure 4C). Note, unlike a distance or a correlation matrix, this is not a symmetric matrix and the diagonals quantify the similarity of response patterns between the conditioning and inference test. To test evidence for representation of auditory-visual or auditory-outcome associations, for each participant 2 RSMs were estimated: one using trials where the correct inference was made during the inference test, and a second using trials where the incorrect inference was made during the inference test (e.g., Figure 4E). Both ‘correct’ and ‘incorrect’ RSMs were then used to estimate the following summary statistics. First, the mean ‘within’ versus mean ‘between’ auditory-visual association was estimated (Figure 4G). Second, we fitted a GLM with 2 EVs to the RSM, to obtain parameter estimates for auditory-visual associations dependent upon (EV 1) or independent upon (EV 2) the value of the associated outcome (Figures S3G–S3I), or obtain parameter estimates for auditory-outcome mappings conditional on (EV 1) or unconditional on (EV 2) sensory cues that predicted the outcome (Figures 6 and S6A–S6C). In both cases, summary statistics were tested at the group level using two approaches: (1) a one-sided Wilcoxon signed-rank test across participants; (2) a one-sided permutation test where the null distribution was generated by estimating the group average 10,000 times, after permuting the identity of all auditory cues in the RSM on each iteration. Correct and incorrect trials were kept separate for this permutation test. For visualization of the group average RSM (Figure 4E), the average correlation coefficient was estimated for each auditory-visual pair for each participant to give a 4x4 matrix.

Using the output t-statistic maps from the third and fourth GLMs, we implemented searchlight RSA (Figures S3C, S6D, and S6E) using a spherical searchlight defined using default settings (Nili et al., 2014): fixed volume of 100 nearest neighbor voxels relative to the center voxel; variable radius with upper limit set to 15 mm to accommodate brain boundaries. The searchlight was swept across each brain volume. Across t-statistic maps (trials), the extracted voxels were correlated using Pearson correlations, and expressed as a correlation coefficient (r). The RSM was then constructed using correctly inferred trials as described for the hippocampal ROI RSA analysis above, and the resulting correlation coefficients were Fisher transformed. A summary statistic was then generated for each searchlight sphere, using the RSM to estimate ‘within’ versus ‘between’ auditory-visual associations (Figures S3C–S3E), ‘within’ versus ‘between’ auditory-outcome associations conditional on the visual cue (Figures 6H–6J), or multiple regression (GLM; see below) to compare different model RSMs (Figures 6E–6G). The summary statistic of interest was then mapped back to the central voxel in the searchlight sphere and saved. The sphere was then shifted and the entire procedure repeated until complete for the entire imaged volume. Across all spheres, this yielded a descriptive map per subject. Across the group, these subject maps were then entered into a second-level random effects analysis.

To compare different model RSMs within the same searchlight sphere (Figures 6E–6G), we used multiple regression (GLM) to compare the Z-scored RSMs across voxels. The GLM included 2 EVs to obtain parameter estimates for auditory-outcome associations that reflected the learned task structure (EV 1) or task-independent value (EV 2) (Figure S6B). For each EV, the regression weight was used as the summary statistic and across all spheres this yielded two descriptive maps per subject, one for each EV.

#### Human fMRI statistics and ROI specification

From the first and second GLMs, we report results at the group-level using whole-brain family wise error (FWE) corrected statistical significance. The cluster defining threshold was p < 0.01 uncorrected and the correct significance level defined as p < 0.05. For univariate effects in the hippocampus, we use an anatomical hippocampal mask (Figure S3A) to extract the raw hippocampal BOLD signal (Figure 4A) and to perform small-volume correction (SVC) for multiple comparisons with FWE peak-level correction at p < 0.05 (Figures 2B and S3D).

All ROIs were defined from contrasts that were orthogonal to the contrasts of interest. To define the seed region for the PPI (Figure 2D), we defined an ROI in bilateral auditory cortex using the contrast between inference test trials and conditioning trials, thresholded at p < 0.001 uncorrected (Figures 2C and S3B). To define an independent hippocampal ROI for RSA, the univariate contrast between correctly inferred and incorrectly inferred trials on the inference test (Figure 2B) was thresholded at p < 0.01 uncorrected (Figure S3F). To define independent masks in medial prefrontal cortex and putative dopaminergic midbrain we used two previous fMRI datasets, reporting functional maps for novel conjunctive representations in medial prefrontal cortex (Barron et al., 2013) and reward prediction error signals in ventral tegmental area (Klein-Flügge et al., 2011) respectively (Figures S6F and S6G). These functional masks were used to perform small-volume correction (SVC) for multiple comparisons with FWE peak-level correction at p < 0.05 (Figures 6H and 6J).
