# Supplementary material for: Neuronal Computation Underlying Inferential Reasoning in Humans and Mice
Source: Cell. 2020 Oct 1;183(1):228–243.e21. doi: 10.1016/j.cell.2020.08.035 (PMC7116148; doi:10.1016/j.cell.2020.08.035)
Supplement: Document S1. Tables S1–S4 [file mmc1.pdf]

Cell, Volume *183*

Supplemental Information

Neuronal Computation Underlying

Inferential Reasoning in Humans and Mice

Helen C. Barron, Hayley M. Reeve, Renée S. Koolschijn, Pavel V. Perestenko, Anna Shpektor, Hamed Nili, Roman Rothaermel, Natalia Campo-Urriza, Jill X. O'Reilly, David M. Bannerman, Timothy E.J. Behrens, and David Dupret

Table S1 | Neural correlates of inference; related to Figure 2.
Brain regions showing significant increase in BOLD signal for correct versus incorrect trials in the inference test (Figure 2B). Statistics reported in the association cortex are FWE whole-brain corrected at the cluster-level. Statistics reported in the hippocampus are peak-level FWE corrected using a small-volume correction method.

Brain region	$P_{FWE\text{-}corr}$	$T_{peak\ level}$	Coordinate		
			x	y	z
**Right Association Cortex**	P<0.001	5.79	66	-42	30
**Left Association Cortex**	P=0.001	5.45	-62	-58	26
**Right Hippocampus**	P=0.022	4.15	32	-26	-4

Table S2 | Functional connectivity during inference; related to Figure 2.
Brain regions showing significant psychophysiological interaction with activity in auditory cortex (seed region) during correct versus incorrect inference (Figure 2D). In all regions, statistics are FWE whole-brain corrected at the cluster-level.

Brain region	$P_{FWE\text{-}corr}$	$T_{peak\ level}$	Coordinate		
			x	y	z
**Cerebellum**	P<0.001	5.63	12	-58	-32
**Visual Cortex**	P<0.001	4.77	20	-90	-10
**Cerebellum**	P<0.001	4.64	-4	-82	-26
**Retrosplenial Cortex**	P=0.012	3.88	2	-44	20
**Retrosplenial Cortex**	P=0.012	3.74	-2	-50	12
**Parahippocampus**	P=0.047	4.43	-18	-30	-18
**Left Hippocampus**	P=0.047	4.12	-22	-30	-6
**Right Hippocampus**	P=0.015	4.23	22	-32	-6
**Parahippocampus**	P=0.015	4.13	24	-24	-26

Table S3 | Representation of the inferred outcome; related to Figure 6.
Brain regions showing significant representational similarity between the auditory cues X_n and associated outcomes Z_n during correct inference trials in the inference test (Figure 6F-G). In all regions, statistics are FWE whole-brain corrected at the cluster-level.

Brain region	$P_{FWE\text{-}corr}$	$T_{peak\ level}$	Coordinate		
			x	y	z
**Dopaminergic Midbrain**	P<0.001	5.56	-2	-18	-20
**medial Prefrontal Cortex**	P=0.003	5.09	8	46	-4
**Retrosplenial Cortex**	P=0.002	4.87	-2	-54	14
**Visual Cortex**	P=0.002	4.76	10	-60	6
**Association Cortex**	P=0.005	4.71	62	2	2

Table S4 | Number of mice; related to Figures 1-7.
Number of mice per group included in the experiment.

Group	Number of mice included per group						
	**Total**	**Used in inference task**	**Implant in dCA1**	**Implant in dCA1, mPFC, VTA**	**Combined electrophysiology-optogenetic silencing**	**ArchT-GFP**	**GFP-control**
**Electrophysiology**	10	5	6	4	1	1	0
**Optogenetic only**	14	14	14	0	0	8	6
